# A social exchange perspective on employee performance in Sierra Leone’s public sector: the roles of authentic leadership, participative decision-making, and trust

**DOI:** 10.3389/fpsyg.2026.1885753

**Published:** 2026-10-15

**Authors:** Ibrahim Sallieu Jalloh, Shixiang Li, Guangjin Zhang, H. M. Wasif Rasheed, Rashid Khurshid

**Affiliations:** 1 School of Public Administration, China University of Geosciences, Wuhan, China; 2 School of Management, Institute of Business Management, Karachi, Pakistan; 3 Institute of Business Management & Administrative Sciences, The Islamia University of Bahawalpur, Bahawalpur, Pakistan

**Keywords:** authentic leadership, employee performance, organizational citizenship behavior, participative decision making, work engagement

## Abstract

**Purpose:**

Based on Social Exchange Theory (SET) and Social Information Processing (SIP), this research attempts to explore the effect that Participative Decision-Making (PDM) has on the performance of employees working in Sierra Leone’s public sector using Affective Trust, Work Engagement, Affective Commitment, and Organizational Citizenship Behavior (OCB) as mediating variables in relation to Authentic Leadership as a moderating variable.

**Design/Methodology/Approach:**

The study used a quantitative and cross-sectional research design. Data collection was conducted among public sector employees in Sierra Leone using a convenience sampling method. The study applied Partial Least Squares Structural Equation Modeling (PLS-SEM) to assess the measurement and structural model hypotheses.

**Findings:**

The empirical results reveal that participative decision-making (PDM) associate with employee performance through affective trust, which associate to work engagement and affective commitment, resulting in organizational citizenship behavior (OCB). The variable of work engagement proved itself as a better predictor of OCB than affective commitment. Besides, authentic leadership moderated the relationship between affective trust and work engagement/affective commitment.

**Originality/value:**

The current research contributes to the SET and SIP frameworks by validating a step-by-step process leading to PDM, which ultimately influences Employee Performance. The study fills one of the significant gaps in the literature by providing evidence from an unexplored area of public sector employees in Sierra Leone.

## 1 Introduction

Employee Performance (EP) is an important factor for organizational effectiveness and quality of public services, but maintaining high levels of employee performance continues to be a major difficulty faced by public sector organizations, especially in developing countries with bureaucratic environments, limited resources, and centralized decisions (Jo and Meier, 2025; Waheed et al., 2025). This kind of environment usually restricts employee autonomy, diminishes motivation, and lowers workplace effectiveness, indicating that factors beyond organizational management affect employee performance (de Oliveira Corrêa et al., 2026; Gruman and Saks, 2011; Tweedie et al., 2019). Evidence from African public-sector settings further indicates that ineffective leadership, limited workforce development, weak employee-manager relationships, and low motivation continue to constrain employee performance and service delivery (Nzimakwe and Utete, 2024). These challenges underscore the need to understand better the organizational mechanisms through which participative management and positive workplace relationships may enhance employee performance in developing public-sector contexts.

These persistent performance challenges suggest that improving employee performance requires more than structural and administrative reforms. Although public-sector reforms have sought to enhance efficiency, accountability, and service delivery, evidence from African public-sector organizations indicates that bureaucratic constraints, limited resources, weak leader-employee relationships, politicized appointments, and inadequate workforce development continue to undermine organizational effectiveness (Ohemeng et al., 2018; Maake et al., 2021; Donkor et al., 2022; Segbenya and Yeboah, 2022; Triansyah et al., 2023). Consequently, scholars have increasingly emphasized the importance of social and behavioral factors in explaining employee performance rather than relying solely on administrative mechanisms (Almquist et al., 2013; McNulty and Ferlie, 2004; Bracci, 2023). However, existing research in African public-sector settings has predominantly examined structural or direct relationships, while giving limited attention to the relational and psychological mechanisms through which participative decision-making, affective trust, authentic leadership, work engagement, affective commitment, and organizational citizenship behavior are associated with employee performance. Addressing this gap, the present study examines these interrelated mechanisms within Sierra Leone’s public sector.

Sierra Leone provides a valuable context for examining these relationships because its public sector operates within a centralized administrative system where limited employee participation may weaken trust, motivation, and ultimately employee performance. Despite the critical role of public-sector employees in national development and service delivery, empirical evidence on participative decision-making, affective trust, and authentic leadership in West African public-sector organizations remains rare, with existing research being heavily dominated by Western and Asian contexts (Ofori and Asare, 2026). Furthermore, prior studies have largely examined direct or single-mediator relationships using cross-sectional designs, while paying limited attention to the sequential relational, motivational, and behavioral mechanisms through which participative decision-making influences employee performance, as well as the contextual role of authentic leadership (Macaspac, 2024; Olowonefa and Musa, 2026). The present study develops and tests a sequential model within Sierra Leone’s public sector to provide a more comprehensive understanding of the mechanisms associated with employee performance, thereby addressing contextual, theoretical, and empirical gaps.

Despite the well-known application of participative decision-making (PDM) as a managerial approach to increase employees’ motivation and performance, empirical findings are inconsistent. Specifically, previous studies have reported positive, insignificant, negative, and null associations between participative decision-making and employee performance. The above inconsistencies point to the necessity to study the internal processes of organizations responsible for PDM effectiveness rather than participation itself. Most prior work focuses on structural and procedural approaches to participative decision-making, without considering the relational and psychological processes underlying the influence of such practices on workers’ performance. Specifically, there is insufficient research on affective trust as a mechanism through which participative decision-making affects employees’ motivational status, although relational exchange is important in the public sector due to low levels of material incentives (Schilke and Lumineau, 2025). Additionally, work engagement and affective commitment are often considered separately, thereby neglecting their distinct contributions to understanding PDM effects on performance (Bakker and Demerouti, 2024).

Existing research provides limited insight into the sequential mechanisms underlying the association between participative decision-making and employee performance. Most previous research focuses either on trust, work engagement, affective commitment, or OCB, and on their relationships with one another, either independently or through the mediation of a single variable (van der Werff et al., 2019; Bakker and Demerouti, 2024). Moreover, leadership is often studied separately as either a mediator or a predictor, without considering that it may influence the relationship between employees’ trust and their motivational state. As a result, there is no information on when trust mechanisms work best, especially for public sector employees (Wang and Liu, 2025). These gaps are especially relevant in African public-sector organizations, where hierarchical structures, resource constraints, and relatively low institutional trust underscore the need to understand the relational, motivational, and behavioral pathways underlying employee performance. Addressing this gap, the present study develops and tests a sequential model that integrates these mechanisms within Sierra Leone’s public sector.

In response to these theoretical, empirical, and contextual gaps, this study examines how participation in decision-making (PDM) is associated with employee performance in Sierra Leone’s public sector, using a sequential, relational, motivational-behavioral framework. Specifically, it investigates the sequential roles of affective trust, work engagement, affective commitment, and organizational citizenship behavior, while examining authentic leadership as a boundary condition influencing the relationships between affective trust and employees’ motivational states. Accordingly, this study is guided by the following overarching research question and objectives. Table 1 presents study-specific objectives:

**Table 1:** Research question and objectives.

**Research Question**	**Research Objective**	**Mechanisms**
How does participation in decision-making (PDM) influence employee performance in the public sector through relational, motivational, and behavioral mechanisms, and under what leadership conditions are these processes strengthened?	To examine how Participative Decision Making (PDM), as a democratic administrative practice, initiates a relational exchange by fostering Affective Trust among civil servants.	The relational foundation
	To investigate how Affective Trust triggers distinct psychological pathways, specifically assessing its capacity to generate both the energetic state of Work Engagement and the emotional state of Affective Commitment.	The motivational activation
	To determine how these internalized motivational states are behaviorally expressed through discretionary Organizational Citizenship Behavior (OCB), and to establish OCB as the critical proximal mechanism driving ultimate Employee Performance in the public sector.	The behavioral translation and performance
	To evaluate how Authentic Leadership functions as a critical boundary condition, specifically testing whether ethical and transparent leadership amplifies the conversion of trust into active engagement and organizational commitment.	The contextual boundary

The integration of the SET and SIP theories into a single theoretical framework will be significant for the current body of theoretical knowledge, as it will allow us to define the motivational and interpretive processes underlying employee performance. The research extends the current body of knowledge by providing an understanding of employee performance through a series of actions involving trust, attitudes, and behavior, as well as the concept of leadership authenticity as a moderating variable. In practice, the results may be useful to public sector leaders/policymakers.

## 2 Theoretical framework and hypothesis development

Theoretically, this study integrates Social Exchange Theory (SET) and Social Information Processing (SIP) to explain how managerial practices shape employee performance. According to SET, workplace relationships follow the principles of reciprocity, according to which positive organizational behavior, along with the support and goodwill expressed through participation in decision-making and genuine leadership, elicits a positive response from employees who reciprocate with positive attitudes and behaviors (Blau, 1964; Cropanzano and Mitchell, 2005). Participatory decision-making in this regard promotes affective trust, which invigorates employees’ motivational states in the form of work engagement and affective commitment, and eventually stimulates discretionary behaviors that facilitate performance. Complementary to this, SIP theory holds that managers and employees define managerial practices and leadership behaviors as social signs to influence their cognitive and emotional reactions and to guide their judgments about appropriate workplace attitudes and behaviors (Salancik and Pfeffer, 1978).

Authentic leadership is an important contextual factor, as it shapes the interpretation of trust cues and participatory actions and, in turn, enhances or diminishes the transfer of trust into engagement, commitment, and citizenship behavior. This framework combines SET and SIP not only to explain why employees return positive treatment with improved performance, but also to describe how social and leadership cues are connected to the interpretive processes by which trust-based relationships lead to the development of performance-sustaining behavior patterns. The hypotheses are formulated below on this theoretical basis. Furthermore, human resource management in the private sector is performance-oriented to maximize profits; in public administration, the conceptualization of Employee Performance (EP) is closely linked to the creation of public value, citizen satisfaction, and the effectiveness of service delivery. The bureaucratic red tape and strict hierarchies in the Sierra Leonean civil service sometimes undermine government employees’ motivation. Thus, the role of Participative Decision Making (PDM) in the research is not only an HR tool but also a democratic approach to administration. Decentralization of power will help public sector organizations to develop internal democratic governments, which are vital for building effective trust to implement complex public policies and surmount institutional inertia. Also, this paper conceptualizes employee performance (EP) as a multistage, relational, and motivational process grounded in participatory practices and leadership context. Involvement in decision-making (PDM) is considered an antecedent that enhances employees’ autonomy to voice, respect, and inclusion, which, in turn, increases affective trust (AT) in the organization and its leadership (Cotton et al., 1988; Mayer et al., 1995). When employees feel that decision-making processes are fair and inclusive, they are more likely to form emotional associations shaped by trust and confidence in organizational intentions, which, in turn, stimulate positive work-related states. On its part, affective trust encourages greater work engagement (WE) and affective commitment (AC) by enhancing employees’ psychological attachment, intrinsic motivation, and the desire to exert discretionary effort in their jobs (Kahn, 1990; Allen and Meyer, 1990). In addition, the above-mentioned motivational states contribute to OCB because employees who are committed to their organization emotionally and are engaged in their job responsibilities volunteer to do more than what they have to in order to support other people working in their organization and the work process (Organ, 1988; Podsakoff et al., 2000). Finally, such discretionary actions can achieve maximum effectiveness in terms of work, service, and performance. In this regard, the term authentic leadership (AL) is introduced as a situational moderator that helps improve the positive influence of affective trust on both work engagement and affective commitment (Avolio and Gardner, 2005; Walumbwa et al., 2008).

The application of the proposed sequential model within Sierra Leone’s public sector is thus a novel and important contribution to the research context. Public administration in developing countries in West Africa, including Sierra Leone, has traditionally been marked by rigid bureaucracy, a large power distance, and centralized power. In such an institutional environment, the use of PDM is not only a standard managerial technique but also a major organizational transformation. When workers, accustomed to a hierarchical environment, are given a chance to participate in decision-making, the cues and obligations that arise in their minds (SIP and SET) can have a greater impact than in Western organizations, where participation has long been established. Moreover, given institutional resource shortages and ongoing civil service reform in Sierra Leone, the investigation of the role that Authentic Leadership plays in turning weak affective trust into actual extra-role behaviors (OCB) is crucial.

### 2.1 PDM and affective trust

A fundamental yet often overlooked dimension of affective trust (AT) in public administration is its built-in element of risk tolerance. By definition, trust involves being willing to be vulnerable to the other side’s behavior (Mayer et al., 1995). In the hierarchical, bureaucratic world of public administration, speaking out, contributing new ideas, or even engaging in additional role behaviors can carry considerable professional risks, including reprimand or exclusion. Affective trust acts as a psychological buffer, increasing an individual’s risk tolerance. The very implementation of PDM implies that people must take the risk of stating their views. If leaders respond positively to their subordinates’ concerns, as in the case of affective trust, employees’ risk perception decreases. Therefore, AT changes employees’ risk calculus in addition to making them ‘happy.’ This allows risk-averse civil servants to engage in discretionary, innovative behavior (OCB) necessary to improve public service provision without fear of bureaucratic punishment.

Drawing on SET, we posit that participative practices shape employee behavior through the principle of reciprocity (Cook et al., 2013; Yin, 2018). PDM is an important source of information and a measure of respect, inclusion, and organizational support; if employees feel that their opinions matter, they perceive this as benevolence on the part of the company, setting in motion social exchange processes beyond formal agreements (Bakker and Demerouti, 2024). Such a phenomenon is especially crucial for the development of affective trust, a measure of emotional connection between employees and their organization, grounded in emotions of care and mutual respect (Ala‘Eddin et al., 2025; Gul et al., 2024). AT is an emotional connection based on feelings of safety and care in social exchanges (Punyatoya, 2019). Contrary to cognitive trust, characterized by transactional reliability, affective trust demonstrates relational characteristics of the employment relation. We claim that PDM builds this type of trust because it shows that employees are treated as equals rather than subordinates, as previous research has shown (Aggarwal et al., 2025).

Moreover, PDM communicates to employees that they are important partners, not agents (Black and Gregersen, 1997). In SET theory, when leaders solicit employee opinions, the high-quality social exchange process begins. Such vulnerability, through which power is vested in subordinates, communicates managerial benevolence and integrity, which are basic antecedents of trust (Mayer et al., 1995). While cognitive trust relies on reliability, affective trust (AT) is an emotional and relational construct. This study hypothesizes that PDM creates a psychologically safe climate for developing AT. Previous literature indicates that participative processes foster greater trust by increasing perceptions of procedural justice and reciprocity (Cotton et al., 1988; Cropanzano and Mitchell, 2005). Hence, we propose that:

*H1*: Participation in decision making is positively related to employees’ affective trust.

### 2.2 Affective trust and employee motivational states

Affective trust does not directly correlate with performance; however, it provides workers with the emotional foundation needed to invest in their roles. Such an investment is reflected in two attitudinal conditions: work engagement and affective commitment. First, work engagement refers to the extent to which employees are vital, committed, and absorbed in work, which frequently goes hand in hand with discretionary effort (Kahn, 1990; Hassan et al., 2024). We claim that when employees trust, they are more likely to feel psychologically secure and to invest their energy and attention in their work, thereby becoming more engaged (Ansong et al., 2025). At the same time, affective trust encourages affective commitment, increasing the degree of attachment and identification of the staff to the organizational goals (Prambudi et al., 2025). As opposed to the short-term orientation associated with engagement, commitment is long-term and makes employees willing to stay in the organization and help achieve success on a large scale, beyond short-term task requirements. Furthermore, trust is not a goal; it is the means of unlocking employees’ potential. Employees who have high affective trust in their bosses are less likely to be obsessed with self-protective observation and are freer to commit their emotional, cognitive, and physical power to the job (Colquitt et al., 2012). On the one hand, trust makes it easier to achieve Work Engagement (WE) - a sense of energy, commitment, and immersion (Kahn, 1990; Schaufeli et al., 2002; Saks, 2006). When employees trust their leaders, they can feel safe enough to lose themselves in the work. Second, trust creates Affective commitment (AC) by fostering personal identification with organizational aims and generating a sense of belonging (Allen and Meyer, 1990; Dirks and Ferrin, 2002). Thus:

*H2a*: Affective trust is positively related to work engagement.

*H2b*: Affective trust is positively related to affective commitment.

### 2.3 From attitudes to behavior: the role of OCB

Based on the Social Exchange Theory (SET) and the Social Information Processing (SIP) theory, this research paper conceptualizes organizational citizenship behavior (OCB) as the result of employees’ motivation processes that rely on trust and information. In a SET approach, employees feel an obligation to reciprocate with favorable treatment when they have positive socio-emotional interactions with their organization, in the form of discretionary, extra-role acts that benefit the organization (Organ, 1988; Podsakoff et al., 2009). Such actions become even more sensitive in the public sector, where quality of service and efficiency play an especially crucial role (Achmad and Zaki, 2025; Etruly and Nafi'ah, 2023), and formal incentives are often subject to restrictions. Supporting the stated perspective, SIP suggests that employees rely on social and organizational indicators to make sense of the appropriate way to respond to workplace behaviors (Salancik and Pfeffer, 1978). Employee engagement and affective commitment are signs from within that an organization is supportive and reliable, and that it deserves additional effort. Such signals shape employees’ thinking, prompting them to go beyond their job descriptions and engage in discretionary behaviors such as helping colleagues and advancing organizational goals (Nguyen and Tran, 2025). Therefore, OCB is the juncture at which attitudinal states, shaped by social exchange relationships and strengthened by contextual information, are transformed into observable work behavior. This study hypothesizes, therefore, that work engagement and affective commitment are among the main attitudinal antecedents that propel OCB as a behavioral manifestation of both reciprocal exchange and a socially constructed sense. Therefore, the hypotheses are:

*H3a*: Work engagement is positively related to organizational citizenship behavior.

*H3b*: Affective commitment is positively related to organizational citizenship behavior.

### 2.4 Moderating role of authentic leadership

Based on the Social Information Processing Theory (SIP), we claim that employees use social cues from their immediate surroundings to explain organizational practices and internal environments (Salancik and Pfeffer, 1978). Leadership behavior is one of the most salient sources of such cues. Although affective trust offers the potential for engagement and commitment, we assume that Authentic Leadership (AL) is a vital boundary condition that triggers this process. True leaders are characterized by self-awareness, relational transparency, balanced processing, and an internalized moral view that gives employees realistic signals that organizational intentions are genuine (Walumbwa et al., 2008; Avolio and Gardner, 2005). We suggest a resource-strengthening interaction in which AL promotes the quality and credibility of trust-based relations. When AL is high, the correlation between affective trust and employees’ attitudes is stronger, as the leader consistently aligns their values with their actions and reinforces psychological safety and meaning (Tobing et al., 2026).

On the other hand, when a leader is not authentic (low AL), the level of trust may not fully translate into engagement because the behavioral cues required to commit to the association are absent. This is supported by recent discoveries indicating that employees tend to perceive more affective trust as a safe base for greater involvement and commitment when they view their leaders as authentic (Lee et al., 2026). Accordingly, we propose:

*H4a*: Authentic leadership positively moderates the relationship between affective trust and work engagement, such that the relationship is stronger when authentic leadership is high.

*H4b*: Authentic leadership positively moderates the relationship between affective trust and affective commitment, such that the relationship is stronger when authentic leadership is high.

### 2.5 Organizational citizenship behavior and employee performance

As a behavioral implication of the motivational and relational employee experience at work, Organizational Citizenship Behavior (OCB) is the culmination of discretionary effort that contributes to broader organizational performance (Organ, 1988). In the public sector context, where formal rewards and punishments are relatively rare, OCB becomes significant for sustaining the quality of service provision and the functioning of the organization through cooperation, proactivity, and problem avoidance (Achmad and Zaki, 2025). People who perceive themselves as being treated supportively and in a trusting manner reciprocate by engaging in voluntary and extra-role activities, going beyond what is required by their official job description and observable in organizational operations (Blau, 1964; Cropanzano and Mitchell, 2005). This is a clear indication of reciprocity rather than fulfilling contractual obligations. On the other hand, the SIP approach indicates that OCB may become a determinant factor in building and sustaining the social environment by signaling cooperation, mutual aid, and collective responsibility, thereby reducing friction among workers (Salancik and Pfeffer, 1978). Even though OCB is conceptually different from task performance, it may facilitate workflow efficiency, service delivery, and task performance by supporting cooperation and coordination among employees, particularly in interdependent public-sector settings (Podsakoff et al., 2009). As the line between extra-role and in-role performance blurs, a larger proportion of employees who uphold citizenship are more likely to be seen and rated as higher performers because of their contributions to the unit’s overall performance. Based on this, OCB can be viewed as the immediate form of behavior that results in better employee performance through positive attitudes and trust-dependent relationships. Therefore, we hypothesize:

*H5*: Organizational citizenship behavior is positively related to employee performance.

### 2.6 Sequential mediation effects

By incorporating the suggested relationships, this research study contributes to creating a sequential pathway in which Participation in Decision Making (PDM) impacts Employee Performance only indirectly, via a chain of psychological and behavioral processes. Based on the Social Exchange Theory (SET), participative practices initially develop Affective Trust, which underlies the psychological situation that is associated with employees’ willingness to reciprocate positive organizational treatment (Blau, 1964; Cropanzano and Mitchell, 2005). Affective Trust (AT) and Employee Performance (EP) cannot be separated in theory, but there must be a line between them. AT is an internal psychological condition- an emotional attachment in terms of safety and mutual care between the civil servant and the agency. On the other hand, EP is an external, behavior-based rating of an employee’s effectiveness in carrying out his or her responsibilities as a public servant. AT stimulates EP because it radically changes the psychological contract; once public employees are exposed to high levels of affective trust, the perceived costs of monitoring and self-protection decrease significantly. This emotional safety frees up cognitive and physical resources, enabling civil servants to focus on productive, task-related work and service delivery rather than on maneuvering through the bureaucratic nervous system. Under conditions of trust, employees are more inclined to experience a unique yet complementary motivational state, i.e., Work Engagement and Affective Commitment, which indicate increased investment in the work role and emotional commitment to the organization (Hassan et al., 2024). These attitudinal states, in turn, are translated into discretionary Organizational Citizenship Behaviors (OCB), in which employees are allowed to work beyond what is stipulated in their jobs. Such citizenship behaviors are aggregated at the behavioral level to achieve greater coordination, improved service quality, and, ultimately, higher Employee Performance (Nguyen and Tran, 2025). In line with the recent empirical evidence, this model places PDM as a distant antecedent, the effects of which can be felt and realized only when it is activated by the proximal levels of psychological states (trust, engagement, commitment) and the performance behaviors (OCB), which is why the model considers the call to unpack the black box between the management practices and performance outcomes (Ansong et al., 2025; Bakker and Demerouti, 2024). Based on the SET logic of reciprocity, therefore, we suggest the following sequential mediation hypotheses:

*H6*: Affective trust, work engagement, and organizational citizenship behavior sequentially mediate the relationship between participation in decision making and employee performance.

*H7*: Affective trust, affective commitment, and organizational citizenship behavior sequentially mediate the relationship between participation in decision making and employee performance.

### 2.7 Hypotheses summary narrative (H1–H7)

Summing up these arguments, this paper presents a theoretically integrated model that describes how participative managerial practices can lead to high employee performance through a systematic chain of relational, attitudinal, and behavioral processes. Based on the Social Exchange Theory, we propose that relationships arising from participation in decision-making (PDM) provide an early signal of respect, inclusion, and organizational support, which, in turn, associate to the development of affective trust (H1). This trust serves as a psychological resource, prompting employees to respond by increasing their work engagement and commitment (H2a, H2b). These motivational conditions, in turn, are expected to be translated into discretionary organizational citizenship behaviors (OCB), through which employees go the extra mile by volunteering rather than performing role duties (H3a, H3b). In realizing the role of context, we also hypothesize that authentic leadership enhances the degree to which affective trust is translated into engagement and commitment by influencing the employee’s interpretation of the participative practices (H4a, H4b). Lastly, OCB is hypothesized to be the most proximal predictor of employee performance, defined as a combination of personal discretionary behaviors that enhance effectiveness and service quality (H5). By integrating these interconnections, we develop a progressive mediation model in which affective trust, motivational conditions (engagement or commitment), and OCB collaborate to mediate the impact of PDM on employee performance (H6, H7).

### 2.8 Theoretical research model

A hypothetical theoretical framework depicting stages of participation in decision-making through employee performance, mediated by affective trust (AT), motivational states (work engagement and affective commitment), and organizational citizenship behavior (OCB), with authentic leadership (AL) moderating the main trust-attitude relations.

Figure 1 presents a multi-stage model that is based on the Social Exchange Theory and supplemented by the Social Information Processing logic. Involvement in the decision-making process is classified as a far organizational practice which associate with the relational exchange by developing affective trust. This trust, in turn, associate with two distinct but complementary motivational pathways: work engagement and affective commitment, which are manifestations of employees’ attachment to their work and to the organization in energetic and emotional terms. These attitudinal states are further enacted behaviorally as organizational citizenship behavior, which is the most proximal antecedent of employee performance. Authentic leadership is an interactive constraint of a situation that serves to increase the transfer of trust into engagement and commitment by cementing the credibility and integrity of signs of participation. Collectively, the model frames employee performance as an accumulation of relational exchanges, motivational activation, and discretionary behavior, rather than as managerial practices in isolation.

**Figure 1:**
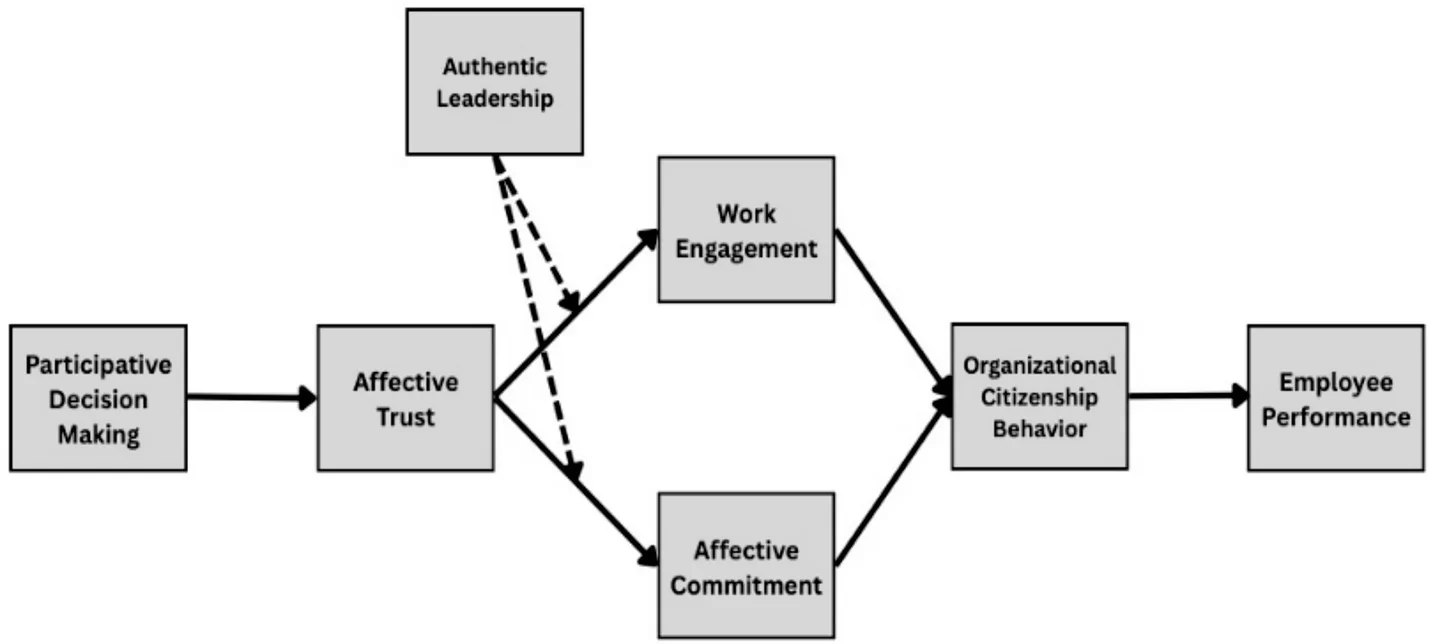
Research model.

### 2.9 Theoretical contributions (sequential mediation focus)

The present study makes three significant theoretical contributions to the body of literature on employee performance and organizational behavior. It contributes to theory by clarifying the mechanisms linking participative decision-making to employee performance through a sequential mediation model. The study goes beyond the prevailing single-stage or direct-effect models, which have yielded inconsistent results in previous studies, by showing that PDM affects performance through a cascading process that includes affective trust, motivational states, and organizational citizenship behavior. Second, the research conceptually separates work engagement and affective commitment as related but independent motivational processes within which trust-based interactions occur, thus responding to recent calls for more fine-grained modeling of employee motivation. Third, by making OCB the final conduit between employees’ attitudes and performance, the research restores behavioral agency to performance models and explains how psychological states are translated into organizational outcomes. Taken together, these contributions enhance social exchange theory by providing an empirical specification of the order, content, and boundary conditions of reciprocal processes that shape employee performance, especially in resource-constrained public sector settings. The Sierra Leonean public sector provides an important theoretical context because hierarchical structures, centralized decision-making, and limited institutional resources increase employees’ reliance on relational and social cues when interpreting participative practices. These institutional characteristics make trust and authentic leadership particularly important mechanisms through which participative decision-making influences employee attitudes and behaviors, thereby extending the application of Social Exchange Theory and Social Information Processing Theory in an underexplored public-sector setting.

## 3 Research methodology

### 3.1 Ethical considerations

Ethical approval was obtained from the Public Service Commission (PSC), Government of Sierra Leone (Ref. No. PSC/ADM/REC/2026/08), dated 08-01-2026. Written informed consent was obtained directly from all participants, and participation was voluntary. Anonymity and confidentiality were maintained, and data were securely stored and used solely for research purposes.

### 3.2 Design and sample

The researchers used cross-sectional data from various public-sector organizations in Sierra Leone. This provided a wide range of representation across age, gender, qualifications, work experience, and supervision details. The population comprises all employees of government-sector organizations across Sierra Leone’s major regions. Although the Sierra Leone government has been making efforts to implement performance management systems, its overall effectiveness is constrained by human capital gaps, structural inefficiencies, and dysfunctional reward systems (Harvey and Bendu, 2021). These institutional issues, aggravated by ineffective leadership and resource scarcity, negatively affect staff involvement and responsibility, and the call to reinforce leadership dedication and investment in psychological resources to foster lasting progress is overwhelming (Kanneh and Haddud, 2016). We argue that the ordered pattern of the relational, attitudinal, and behavioral processes that can affect employee performance can be moderated by managers’ leadership styles in public sector organizations (Jensen et al., 2016). The research was conducted in 08 departments of the Sierra Leonean public institutions. To capture a diverse cross-section of the civil service, data were collected from eight key public-sector departments in Sierra Leone. The distribution of our final sample (*N* = 431) and the total population of senior employees (administrators) in these public sector organizations were 644, using non-probability convenience sampling. Data were gathered among employees of the Sierra Leonean public sector using a structured questionnaire. To ensure a high coverage rate, the survey was also shared on social media (WhatsApp, Facebook) and in personal circles via direct links and QR codes. Consequently, researchers argue that the sample provides a reasonable and functional representation of the broader working conditions and attitudes within Sierra Leone’s public sector, even if strict probabilistic representation cannot be claimed. In all, 538 paper-and-pencil questionnaires were distributed purposively to permanent and senior administrative staff over 1 month, using a drop-off-and-pick-up procedure. Four hundred seventy questionnaires, representing 87.4%, were returned, but after missing-value and outlier analysis, 431 (80.11%) were deemed usable. The descriptive statistics are shown in Table 2.

**Table 2:** Measurement scales of constructs.

**Construct**	**Items**	**Items details with modifications**	**Source**
Participation in decision making (PDM) – 5 items	PDM1	I influence how my daily work tasks are carried out.	Siegel and Ruh (1973)
	PDM2	I influence the methods or procedures used in my job.	
	PDM3	I have influence over decisions about my immediate work environment (e.g., tools, equipment, workspace).	
	PDM4	I influence the scheduling and allocation of my work assignments.	
	PDM5	I influence broader decisions that affect my role within the organization.	
Affective trust (AT) – 5 items	AT1	“We have a sharing relationship. We can both freely share our ideas, feelings, and hopes.”	McAllister (1995)
	AT2	“I can talk freely to this individual about difficulties I am having at work and know that (s)he will want to listen.”	
	AT3	“We would both feel a sense of loss if one of us were transferred and we could no longer work together.”	
	AT4	“If I shared my problems with this individual, I know he would respond constructively and caringly.”	
	AT5	“I have to say that we have made a considerable emotional investment in our working relationship.”	
Work engagement (WE) – 9 items	WE1	“At my work, I feel bursting with energy.”	Schaufeli et al. (2006)
	WE2	“At my job, I feel strong and vigorous.”	
	WE3	“When I get up in the morning, I feel like going to work.”	
	WE4	“I am enthusiastic about my job.”	
	WE5	“My job inspires me.”	
	WE6	“I am proud of the work that I do.”	
	WE7	“I feel happy when I am working intensely.”	
	WE8	“I get carried away when I am working.”	
	WE9	“I am immersed in my work.”	
Affective commitment (AC) – 6 items	AC1	I would be very happy to spend the rest of my career with this organization.	Meyer et al. (1993)
	AC2	I really feel as if this organization’s problems are my own.	
	AC3	I feel a strong sense of “belonging” to my organization.	
	AC4	I feel “emotionally attached” to this organization.	
	AC5	I feel like “part of the family” at my organization.	
	AC6	This organization has great personal meaning for me.	
Organizational citizenship behavior (OCB) – 14 items	OCB1	I attend non-mandatory meetings that are important for improving public service delivery.	Lee and Allen (2002)
	OCB2	I keep myself updated on new government policies and administrative developments.	
	OCB3	I follow informal workplace norms that help maintain order and efficiency in my department.	
	OCB4	I defend my ministry/agency when others unfairly criticize its work.	
	OCB5	I am concerned about my organization’s public image.	
	OCB6	I take extra time to complete tasks that benefit the effectiveness of the public sector.	
	OCB7	I volunteer for additional duties that contribute to my organization’s success.	
	OCB8	I willingly help colleagues who have heavy workloads.	
	OCB9	I assist coworkers who have been absent so that work continues smoothly.	
	OCB10	I go out of my way to help new employees adjust to the public sector environment.	
	OCB11	I take time to listen to coworkers’ concerns and challenges.	
	OCB12	I help orient new staff members even when it is not part of my formal duties.	
	OCB13	I willingly give time to help colleagues with work-related problems.	
	OCB14	I support coworkers in resolving workplace conflicts and challenges.	
Authentic leadership (AL) -16 items	AL1	My supervisor clearly expresses what he/she truly means.	Walumbwa et al. (2008)
	AL2	My supervisor openly acknowledges his/her own mistakes when they occur.	
	AL3	My supervisor encourages everyone to voice their honest opinions.	
	AL4	My supervisor is transparent about difficult truths.	
	AL5	My supervisor expresses genuine emotions that are consistent with his/her feelings.	
	AL6	My supervisor’s actions are consistent with his/her stated beliefs.	
	AL7	My supervisor makes decisions guided by their core values.	
	AL8	My supervisor encourages me to take stands that are consistent with my core values.	
	AL9	My supervisor adheres to high ethical standards when making difficult decisions.	
	AL10	My supervisor actively seeks out views that challenge his or her deeply held positions.	
	AL11	My supervisor analyzes all relevant data before reaching a decision.	
	AL12	My supervisor carefully listens to diverse points of view before forming a conclusion.	
	AL13	My supervisor actively seeks feedback to improve his/her interactions with others.	
	AL14	My supervisor accurately understands how others perceive his/her capabilities.	
	AL15	My supervisor recognizes when it is necessary to reevaluate his/her position on important issues.	
	AL16	My supervisor demonstrates an understanding of how his/her actions affect others.	
Employee performance (EP)	EP1	I adequately complete assigned duties.	Williams and Anderson (1991)
	EP2	I fulfill the responsibilities specified in my job description.	
	EP3	I perform the tasks expected of me.	
	EP4	I meet the formal performance requirements of the job.	
	EP5	I engage in activities that directly positively affect my performance evaluation.	
	EP6	I conscientiously perform all aspects of the job I am obligated to do.	
	EP7	I consistently perform all essential duties of my role.	

Using PLS-SEM in this study is methodologically sound, as most experts view it as highly suitable for testing a conceptual model comprising many constructs, as well as mediation and moderation effects (Hair et al., 2012; Henseler et al., 2009). There are several reasons why one should use SEM-PLS in this study. First, some scholars from the social sciences, such as management, marketing (Hair et al., 2012), human resource management, business research (Hair et al., 2014), and MIS, are applying it. Second, it aids in theory testing (Hair et al., 2014). Third, PLS-SEM can be easily applied to small sample sizes and measurement scales. Finally, this method is especially beneficial for this study, as it is statistically flexible, (1) the prediction-oriented objective of the framework, (2) high theoretical model complexity involving multi-path sequential mediation and moderation, and (3) latent construct composite evaluation (Hair et al., 2014). To address the potential for Common Method Bias (CMB) inherent in self-reported survey data, this study has employed both procedural and statistical controls, as suggested by Podsakoff et al. (2003). Specifically, procedurally, measures were incorporated into the research design by maintaining total anonymity and confidentiality for all participants, which helps reduce evaluation apprehension and social desirability biases. Finally, questionnaire items were worded clearly to avoid leading questions. Once the data were collected, a complete collinearity analysis was performed using Smart-PLS, an extensively used and highly robust approach for detecting Common Method Bias in variance-based Structural Equation Modeling (SEM) (Kock, 2015). As suggested by Kock (2015), a Variance Inflation Factor (VIF) value above 3.3 for any latent construct indicates pathological collinearity and CMB danger. In the current research. Another researcher O’brien, 2007 describe that VIF values below 5 are generally considered indicative of an acceptable level of multicollinearity. Following Kock (2015), a full-collinearity test was performed across all latent constructs. The full-collinearity VIF values ranged from 1.821 to 3.362 (Work Engagement = 3.362, Participative Decision-Making = 2.145, Affective Commitment = 2.810, Authentic Leadership = 2.451, Organizational Citizenship Behavior = 2.912, Employee Performance = 3.120). While Work Engagement’s VIF (3.362) slightly exceeds the conservative 3.3 threshold, it remains well within acceptable limits < 5.0 (O’brien, 2007). Additionally, Harman’s single-factor test was conducted by executing an unrotated principal component analysis on all measurement items. The analysis yielded 7 distinct factors with eigenvalues greater than 1.0, with the first factor accounting for 34.2% of the variance, well below the critical 50% cutoff. These results indicate that common method bias does not pose a significant threat to the validity of the structural model.

### 3.3 Instrumentation and measures

In this research, standard scales that have high reliability and validity were used. The questionnaire was divided into two parts. The research constructs were first presented, followed by the respondents’ demographics. The second segment used a 62-item Likert scale to collect responses. The translation-backtranslation process was used to ensure accuracy and clarity of items to the Sierra Leone cultural setting. The back-to-back translations will help us complete our questionnaire (see Table 2). The consistency of the measurement items was assessed using Cronbach’s alpha. Certain changes to the scale items’ descriptions were made based on participants’ feedback and recommendations to improve readability and understanding. We also ensured that the questionnaire’s scale items were appropriately described and phrased for the participants.

The scales used in the current research were based on well-known, widely used scales to ensure reliability and validity, and all items were rated on a 5-point Likert scale from 1 (strongly disagree) to 5 (strongly agree). The six-item scale of Siegel and Ruh (1973) was used to evaluate Participative Decision-Making (PDM), which included the following question: “I have influence over how my daily work tasks are carried out.” The five-item scale developed by McAllister (1995) was used to measure *Affective Trust (AT)*. This tool was chosen precisely to elicit the emotional aspect of trust; it addresses interpersonal care and emotional attachment rather than reliability or competence (Ng, 2023). Such samples would involve, “I can talk freely to this individual about difficulties at work and know that he/she will want to listen.”

The scale of *Work Engagement (WE)* was measured using the Utrecht Work Engagement Scale (UWES-9), a 9-item scale developed by Schaufeli et al. (2006). The short version was chosen for its strong psychometric properties and high potential to measure vigor, dedication, and absorption (e.g., “In my work, I feel like I am bursting with energy”). The scale used to measure Affective Commitment (AC) was a six-item scale by Meyer et al. (1993), in which negative statements were reworded (e.g., “I feel a strong sense of belonging to my organization”). Organizational Citizenship Behavior (OCB) was measured using a 14-item scale by Lee and Allen (2002). An example of an OCB statement is, “I defend my ministry/agency from unjust criticism from others.” The Authentic Leadership (AL) measure was based on the 16-item Authentic Leadership Questionnaire (ALQ) of Walumbwa et al. (2008), which includes aspects of self-awareness, relational transparency, internalized moral perspective, and balanced processing (“My supervisor can describe others’ perceptions about his/her abilities.”). Employee Performance (EP) was based on the 7-item In-Role Behavior scale of Williams and Anderson (1991). Negatively stated items were reformulated (“I perform my tasks appropriately”).

### 3.4 Data analysis

Partial Least Squares Structural Equation Modeling (PLS-SEM) was selected as the analytical technique using SmartPLS 4 based on key methodological criteria (Hair et al., 2021). First, the theoretical objective of this study is prediction-oriented, seeking to explain endogenous drivers of employee attitudinal and behavioral outcomes. Second, the framework exhibits high structural complexity, incorporating sequential multi-path mediation alongside moderation. PLS-SEM is particularly well-suited for estimating complex conceptual models without imposing strict parametric assumptions on the underlying structural distributions. In addition, PLSpredict was employed to evaluate the model’s out-of-sample predictive performance, while making PLS-SEM an appropriate analytical approach for the objectives of this study (Hair et al., 2011, 2017, 2019; Ringle et al., 2015; Shmueli et al., 2019). Prior to data analysis, data screening was conducted on the final sample (N = 431). No missing values were present, as incomplete questionnaires were excluded during the initial manual screening phase prior to data entry; thus, no imputation procedures were required or performed. Multivariate outliers were examined using Mahalanobis distance evaluated at *p* < 0.001. No multivariate outliers were detected in the dataset (0 cases removed). The absence of extreme outliers is attributed to the bounded 5-point Likert scale (1 = Strongly Disagree to 5 = Strongly Agree) used across all measurement items, which restricts extreme score variance. Construct reliability and convergent and discriminant validity were evaluated through confirmatory factor analysis. Hypothesized direct, mediating, sequential mediating, and moderating relationships were tested in the structural model. Supplementary analyses, including missing value imputation, outlier detection, correlation analysis, and descriptive analysis, were conducted using IBM SPSS 23.0 to examine the direction and strength of relationships and to assess the robustness of the findings.

Although demographic and work-related characteristics (gender, age, education level, organizational tenure, and supervisor tenure) were recorded to describe the sample profile, they were intentionally excluded as control variables in the structural model based on established methodological and theoretical grounds. Methodological scholars strongly caution against uncritically including demographic controls without a compelling theoretical baseline, as impotent control variables can unnecessarily consume degrees of freedom, reduce statistical power, and potentially distort parameter estimates. Because our conceptual framework focuses specifically on proximal psychological and behavioral mechanisms grounded in Social Exchange Theory and Social Information Processing theory, where structural practices and relational perceptions exert a far more direct influence on performance than distal demographic traits, omitting these controls preserves structural model parsimony and ensures parameter interpretability (Hair et al., 2021).

## 4 Results

The suggested model is based on Partial Least Squares Structural Equation Modeling (PLS-SEM) to investigate the predictive and associations among variables. Based on the Social Exchange Theory, this paper proposes a sequential process in which relational resources (Participative Decision-Making and Authentic Leadership) facilitate the development of affective trust. This confidence then increases employees’ attitudinal (Work Engagement and Affective Commitment) outcomes, which eventually lead to behavioral (Organizational Citizenship Behavior and Employee Task Performance) outcomes. The technique can robustly estimate path coefficients, cross effects, and interaction terms, thereby enabling theoretical validation and prediction. Although the relationship between Social Exchange Theory and this model is reciprocal, Social Information Processing (SIP) theory is complementary, providing insight into how employees perceive leadership behaviors. Authentic Leadership, according to SIP, and Participative Decision-Making are important social cues. Employees interpret these cues in order to create a reality of Affective Trust. This optimism about the work environment then gradually influences their attitudes (Engagement, Commitment) and behaviors (Performance, OCB).

The present chapter is written in the following sequence: data screening and the provision of respondent profiles are performed first, followed by preliminary analyses, such as the response rate. Also, data coding, missing data, data normality, collinearity diagnostics, and outliers are discussed in this chapter. In addition, the researcher conducted the primary analysis of the data after a rigorous screening process, beginning with a measurement model analysis to ensure that the modified measures were suitable. A measurement model analysis is conducted to assess the discriminant validity, convergent validity, internal consistency reliability, and item reliability of the altered constructs. Moreover, the structural analysis is conducted and presented in terms of the R-square value, the model’s predictive significance, and the importance of variables in testing hypotheses, based on data collected with the instrument.

### 4.1 Demographic analysis

Table 3 has the demographic profile of the final sample (*N* = 431). The majority of respondents were men (82.8 percent) and fairly young (66.1 percent were aged 18–30). The study acknowledges the gender imbalance in the sample, and this distribution reflects context-specific constraints in Sierra Leone, where differences in digital and internet access, participation in survey-based research, and socio-cultural factors can influence response patterns. Similar gender disparities in survey participation and access to digital information have been reported in prior studies conducted in developing-country contexts (Ofori et al., 2021). It is interesting to note that the sample is quite highly qualified, with more than 85 percent of respondents holding a Bachelor’s or Master’s degree (59.2 percent Bachelor’s and 26.7 percent Master’s). This indicates a highly educated workforce with cognitive preparedness and professional anticipation of Participative Decision-Making, further underlining the applicability of PDM as a vital source of relational input in this regard. The theoretically important gap between organizational and dyadic experience was examined in terms of tenure. Although the sample is well-organized, the majority (60.6%) have served 5–10 years, and tenure in the dyad with the current supervisor is relatively short (60.8% serving with their current leader for 3 years or less). This tenure gap is critical according to the Social Exchange Theory. It suggests that although employees are fixed within the organization (system), their relational contract with particular leaders is still in its initial stages. The context, in particular, makes the role of Authentic Leadership and the creation of Affective Trust even more significant, as it is necessary to stabilize the exchange relationship in more recent dyads where historical trust has not yet developed.

**Table 3:** Study demographic information.

**Demography**	**Mean**	**SD**	**Description**	**No of responses**	**Percentage**
Gender	1.83	0.378	Female	74	17.2%
			Male	357	82.8%
Age	1.56	0.897	18–30 years	285	66.1%
			31–40 years	78	18.1%
			41–50 years	42	9.7%
			Above 50 years	26	6.0%
Education	2.50	1.642	Certificate / Diploma	17	3.9%
			Bachelor’s degree	255	59.2%
			Master’s degree	115	26.7%
			Above Master	44	10.2%
Tenure	3.24	1.171	<1 Year	55	12.8%
			1–2 Years	77	17.9%
			3–4 Years	24	5.6%
			5–10 Years	261	60.6%
			>10 Years	14	3.2%
Years served under the current boss	2.43	1.225	<1 Year	109	25.3%
			1–3 Years	153	35.5%
			4–5 Years	80	18.6%
			6–10 Years	53	12.3%
			>10 Years	36	8.4%

### 4.2 Data coding

The information was then also coded and entered into SPSS V23 after careful review. Coding of data (Table 4) was performed in the SPSS Variable View page, and each variable was coded using the series of letters that form its name. Again, each item was assigned a number depending on its position in the questionnaire.

**Table 4:** Variable coding.

**Variables**	**Code**
Participation in decision making	PDM
Affective trust	AT
Work engagement	WE
Affective commitment	AC
Organizational citizenship behavior	OCB
Authentic leadership	AL
Employee performance	EP

#### 4.2.1 Data screening

Consequently, Hair et al. (2010) state that incomplete questionnaires were excluded from the data analysis. During data screening, 39 of the 470 surveys were found to be invalid due to insufficient responses. Consequently, Hair et al. (2010) indicate that such incomplete surveys were not accounted for in the study. After the screening process, 431 questionnaires remained for further analysis; this number is sufficient (Hair et al., 2010). Incomplete questionnaires were removed during initial screening prior to data entry. The final analyzed dataset (*N* = 431) contained zero missing values. Outliers were also detected and treated in this study. Multivariate outliers were examined using Mahalanobis distance evaluated at *p* < 0.001. According to Tabachnick and Fidell (2013), the Mahalanobis distance was also used to identify and correct multivariate outliers. No multivariate outliers were detected in the dataset (0 cases removed). The absence of extreme outliers is attributed to the bounded 5-point Likert scale (1 = Strongly Disagree to 5 = Strongly Agree) used across all measurement items, which restricts extreme score variance.

The researcher also adopted the numbering of responses used by Hair et al. (2010).

### 4.3 Expectations of fundamental statistics

Following the screening, the data were checked for several basic statistical conditions associated with the research variables, including multicollinearity and normality, to verify the findings and correct simple errors such as Type I and Type II errors.

#### 4.3.1 Multicollinearity analysis

When the correlation between an independent variable and the other independent variables is insignificant, a test (Table 5) is used to evaluate the relationship between two or more exogenous variables (Hair et al., 2010). In addition, according to Tabachnick and Fidell (2013), the identification of variance inflation factors (VIFs) and tolerance values is the most reliable statistical test for detecting multicollinearity, particularly when the independent variables are highly correlated.

**Table 5:** Multicollinearity analysis (dependent variable: EP).

**Model**	**Collinearity Statistics**	
	**Tolerance**	**VIF**
PDM	0.391	2.556
AT	0.351	2.852
AC	0.384	2.601
WE	0.397	3.362
OCB	0.341	2.930
AL	0.630	1.587

Tabachnick and Fidell (2013) provide the traditional levels of tolerance (above 0.2) and VIF (below 5). As seen in Table 5 above, all variables have tolerance values greater than 0.2 and VIFs less than 5, indicating no multicollinearity. However, Table 6 shows that a correlation matrix was generated in SPSS to assess multicollinearity among the individual latent constructs. The data also suggest that none of the independent latent constructs are significantly related; in other words, all the data in the correlation matrix are below the critical value (*r* = 0.9). This indicates a lack of a strong association between the variables (Hair et al., 2010).

**Table 6:** Correlation analysis.

Variables		PDM	AT	AC	WE	OCB	EP	AL
PDM	Pearson correlation	**1**						
	*N*	**431**						
AT	Pearson correlation	0.717	**1**					
		0.000						
	*N*	431	**431**					
AC	Pearson correlation	0.367	0.561	**1**				
		0.000	0.000					
	*N*	431	431	**431**				
WE	Pearson correlation	0.638	0.644	0.683	**1**			
		0.000	0.000	0.000				
	*N*	431	431	431	**431**			
OCB	Pearson correlation	0.610	0.694	0.622	0.758	**1**		
		0.000	0.000	0.000	0.000			
	*N*	431	431	431	431	**431**		
EP	Pearson correlation	0.372	0.399	0.268	0.401	0.588	**1**	
		0.000	0.000	0.000	0.000	0.000		
	*N*	431	431	431	431	431	**431**	
AL	Pearson correlation	0.177	0.312	0.594	0.454	0.409	0.166	**1**
		0.000	0.000	0.000	0.000	0.000	0.001	
	*N*	431	431	431	431	431	431	**431**

#### 4.3.2 Normality analysis

According to Kline (2011), the data indicate a more significant issue, as the skewness and kurtosis values exceed 3 and 10, respectively (see Table 7). However, current research follows the guidelines of Hair et al. (2010). If the variables’ values exceed ±2.58, they should be considered to have violated the normality assumption.

**Table 7:** Skewness and Kurtosis results.

Variables	**Skewness**		**Kurtosis**	
	Statistic	Std. Error	Statistic	Std. Error
PDM	−0.637	0.118	−0.259	0.235
AT	−0.717	0.118	0.017	0.235
AC	−0.259	0.118	−0.348	0.235
WE	−0.215	0.118	−0.662	0.235
OCB	−0.431	0.118	−0.480	0.235
EP	−1.215	0.118	1.791	0.235
AL	−0.790	0.118	−0.072	0.235

The skewness and kurtosis results from the normality test indicate that each variable satisfies the normality assumption. According to Hair et al. (2010), skewness and kurtosis values in Table 6 range from −0.215 to −1.215 and −0.017 to 1.791, respectively, and are all less than ±2.58.

#### 4.3.3 Descriptive statistics of the study

The descriptive statistics for the major variables, including minimum, maximum, and standard deviation, are shown in Table 8. The study variables include mean scores ranging from 3.47 to 4.18 and standard deviations ranging from 0.7241 to 0.9904.

**Table 8:** Descriptive stats of the data.

Variables	*N*	Minimum	Maximum	Mean	Std. Deviation
PDM	431	1.00	5.00	3.9095	0.87661
AT	431	1.40	5.00	3.7903	0.87221
AC	431	1.00	5.00	3.5352	0.87714
WE	431	1.25	5.00	3.5832	0.83839
OCB	431	1.33	5.00	3.7970	0.82995
EP	431	1.00	5.00	4.0272	0.74302
AL	431	1.00	5.00	3.6679	0.99214

### 4.4 Assessment of PLS-SEM path model

To achieve the research objectives, the second section of the research results uses PLS-SEM to test the theoretical assumptions (Hair et al., 2012). Henseler and Sarstedt (2013) prefer applying PLS-SEM to test the postulated relationships among endogenous and Exogenous latent constructs and to visualize them with the help of path diagrams, as goodness-of-fit indices cannot be considered an appropriate or adequate method for testing a study model. Henseler et al. (2009) and Chin (1998) indicate that data analysis is performed in two basic procedures (Figure 1) to evaluate the PLS-SEM path: the measurement model and the structural model (Figure 2).

**Figure 2:**
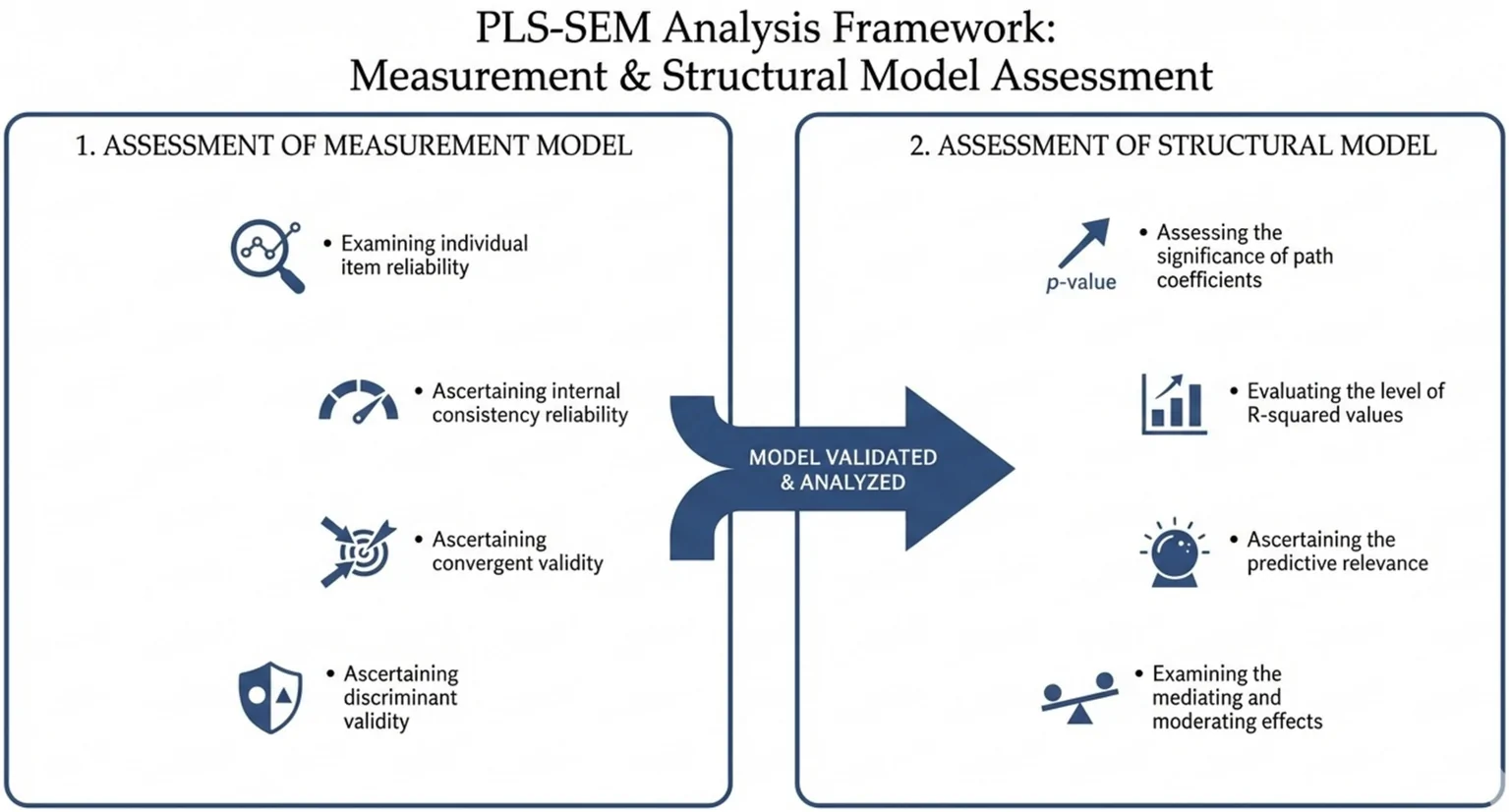
PLS-SEM path assessment model.

### 4.5 Measurement model

The measurement model (Figure 3) included assessing the reliability and convergent and discriminant validity of the constructs. Before running reliability and validity tests, the researcher also analyzed the factor loadings of every construct of the study variables. As recommended by Hair et al. (2014), factors with loadings below 0.50 were omitted from the analysis because they were not believed to make a significant contribution to the study. The results in Table 9 indicate that all items in the model have an outer loading of 0.5 or higher.

**Figure 3:**
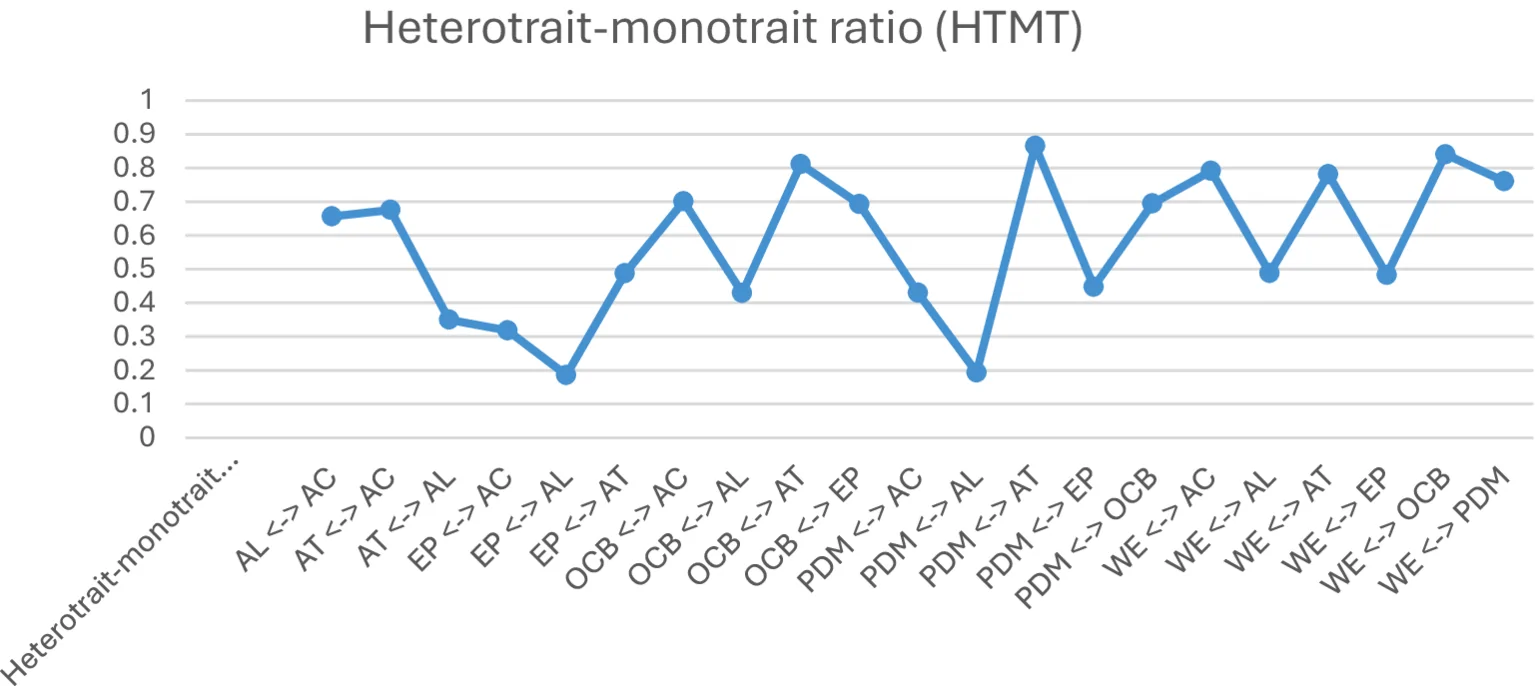
Heterotrait-monotrait ratio (HTMT).

**Table 9:** Reliability and validity results of research model.

**Constructs**	**Items**	**Loadings**	**CBA**	**Composite reliability (rho_a)**	**AVE**
Participation in decision making (PDM)	PDM1	0.739	0.834	0.841	0.596
	PDM2	0.768			
	PDM3	0.697			
	PDM4	0.834			
	PDM5	0.814			
Affective trust (AT)	AT1	0.722	0.807	0.810	0.572
	AT2	0.670			
	AT3	0.856			
	AT4	0.650			
	AT5	0.856			
Work engagement (WE)	WE1	0.754	0.858	0.862	0.501
	WE2	0.695			
	WE3	0.755			
	WE4	0.718			
	WE6	0.678			
	WE7	0.676			
	WE8	0.726			
	WE9	0.655			
Affective commitment (AC)	AC1	0.827	0.844	0.842	0.564
	AC2	0.828			
	AC3	0.753			
	AC4	0.698			
	AC5	0.698			
	AC6	0.686			
Organizational citizenship behavior (OCB)	OCB1	0.692	0.912	0.912	0.508
	OCB2	0.630			
	OCB3	0.738			
	OCB4	0.739			
	OCB5	0.685			
	OCB6	0.743			
	OCB7	0.763			
	OCB8	0.737			
	OCB10	0.716			
	OCB11	0.723			
	OCB12	0.680			
	OCB14	0.695			
Authentic leadership (AL)	AL1	0.742	0.952	0.961	0.609
	AL2	0.754			
	AL3	0.736			
	AL4	0.744			
	AL5	0.797			
	AL6	0.803			
	AL7	0.817			
	AL8	0.820			
	AL9	0.811			
	AL10	0.780			
	AL11	0.802			
	AL12	0.811			
	AL13	0.766			
	AL14	0.731			
Employee performance (EP)	EP1	0.711	0.844	0.856	0.512
	EP2	0.658			
	EP3	0.669			
	EP4	0.682			
	EP5	0.772			
	EP6	0.751			
	EP7	0.757			

The measurement model initially comprised 62 items. Following the removal of five items based on the measurement model assessment, the final measurement model retained 57 items for construct measurement. The literature (Hair et al., 2011) recommends that the acceptable value of average variance extracted (AVE) should be at least 0.5, and that the acceptable value of composite reliability (CR) should be at least 0.7. The variables in Table 8 and Figure 4 are all highly reliable, and the value of the AVE of every variable is above the 0.50 cutoff mark, which suggests that the measurement model is reliable enough to be further analyzed. The measurement model was analyzed to ensure that the outer loadings were sufficient to demonstrate the indicators’ reliability and convergent validity. Based on the instructions of Hair et al. (2019), items with loadings below the suggested value of 0.708 and in the gray area (0.40–0.7) were considered problematic; those with loadings below 0.50 were defined as problematic. As a result of this, five items (WE5 = 0.481, OCB9 = 0.433, OCB13 = 0.447, AL15 = 0.428, and AL16 = 0.486) were dropped from the scale. The omission of these factors led to a slightly increase in both Composite Reliability (CR) and Average Variance Extracted (AVE), thereby ensuring the model met the standards for construct validity.

**Figure 4:**
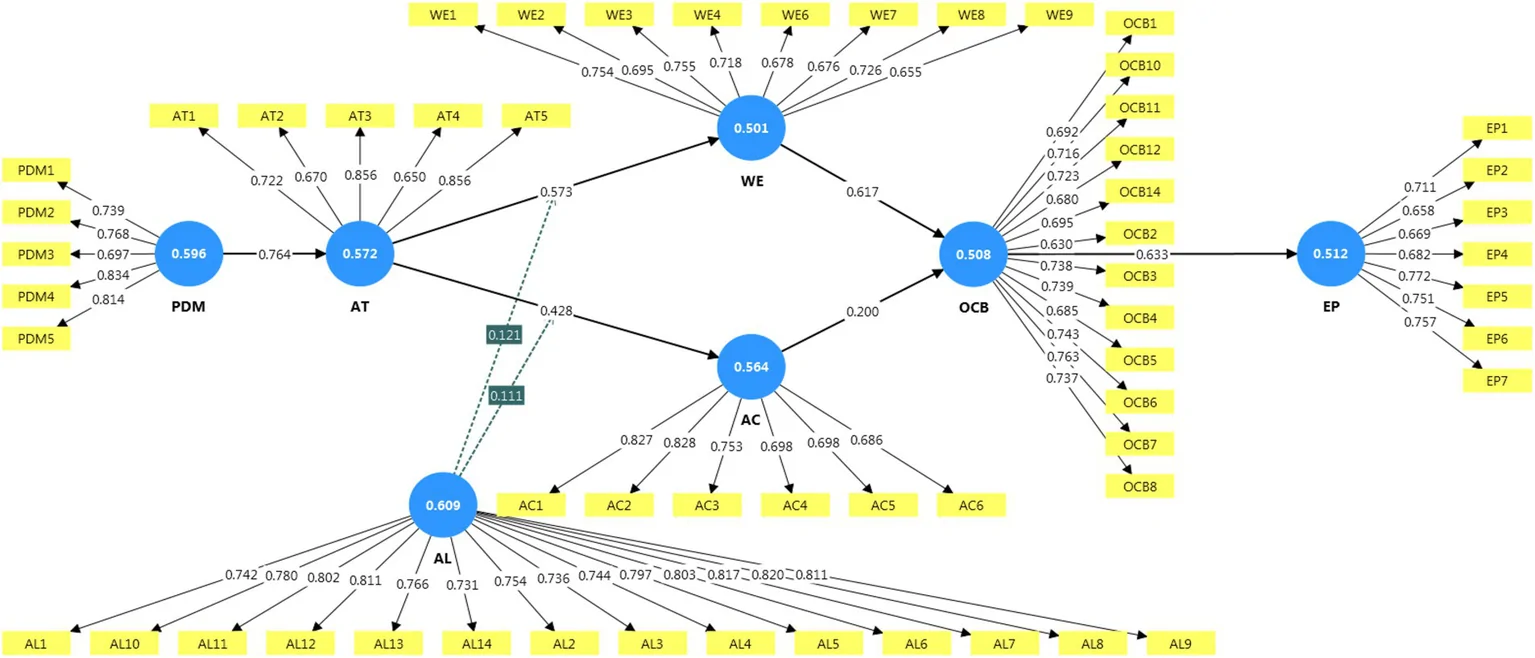
AVE and factor loadings of all variables with path coefficient.

To confirm that the variables are internally consistent, Cronbach’s alpha is also computed. According to George and Mallery's (2003) general guideline, values greater than 0.9, 0.8, and 0.7 are categorized as excellent, good, and acceptable, respectively. Figure 5 shows the CBA value, the Lodgings item, and the Path coefficients.

**Figure 5:**
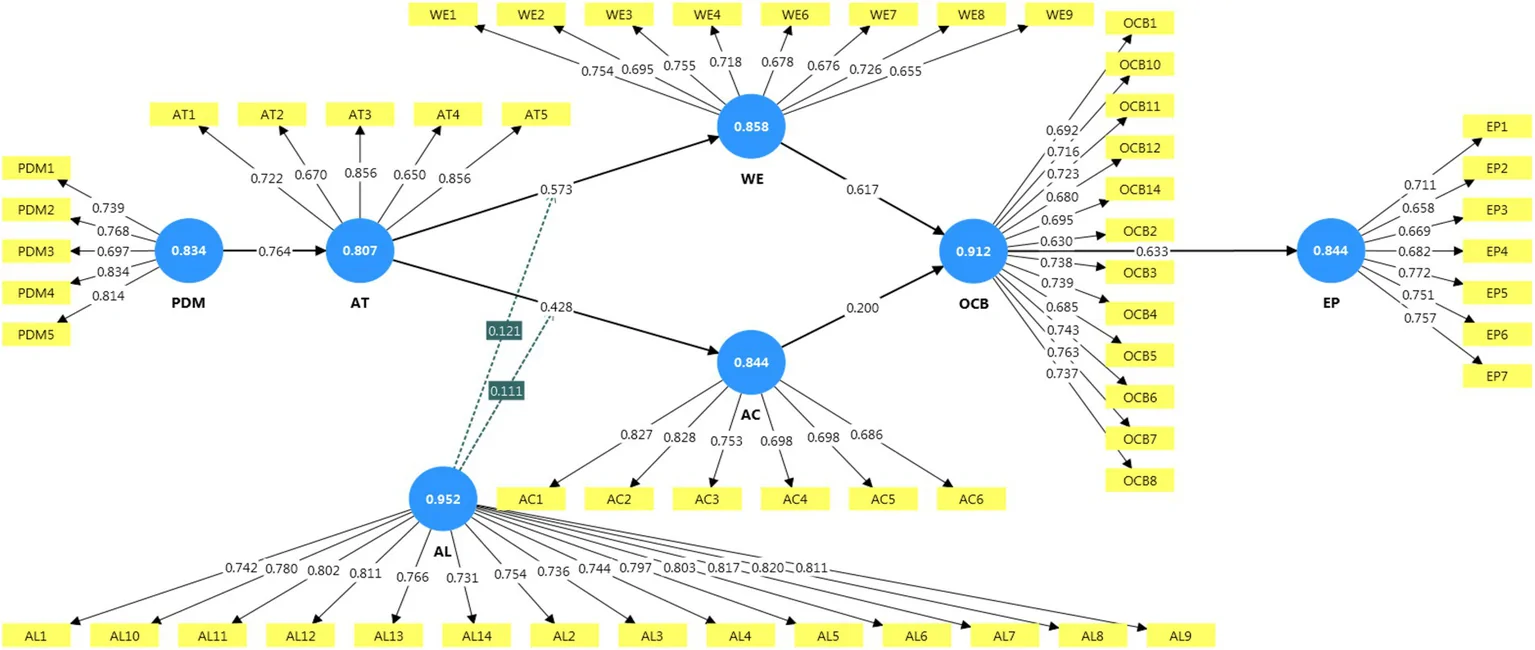
Factor loadings and CBA value of all variables with path coefficient.

#### 4.5.1 Model discriminant validity

Farrell and Rudd (2009) elaborate that discriminant validity is the extent to which a latent variable differs from the other latent variables. The research’s AVE (0.5 or above) indicates discriminant validity per the norms established by Fornell and Lacker (1981). Discriminant validity can be assessed by examining the correlations between the latent variables and the square roots of their AVEs.

As reported in Table 10, while the Fornell–Larcker criterion showed close correlations between Participative Decision-Making (PDM) and Affective Trust (*r* = 0.764), as well as Work Engagement (WE) and OCB (*r* = 0.756), modern PLS-SEM literature notes that Fornell–Larcker exhibits low sensitivity in detecting discriminant validity issues compared to HTMT (Henseler et al., 2015). Conceptually, PDM refers to structural and behavioral opportunities provided to employees to participate in decision-making, whereas Affective Trust reflects an employee’s emotional reliance on and faith in leadership and peers. While high PDM fosters trust, the two remain distinct construct domains (input structure vs. relational psychological state). Similarly, Work Engagement represents a affective-cognitive state of vigor, dedication, and absorption during core task performance, whereas Organizational Citizenship Behavior (OCB) represents voluntary, discretionary behaviors that go beyond formal job duties to support the organization. Cross-loading analysis confirmed that all individual indicator items loaded highest on their targeted construct without significant cross-loadings.

**Table 10:** Model discriminant validity (Fornell-Larcker criterion).

Variables	AC	AL	AT	EP	OCB	PDM	WE
AC	**0.751**						
AL	0.646	**0.780**					
AT	0.575	0.332	**0.756**				
EP	0.293	0.170	0.414	**0.716**			
OCB	0.628	0.435	0.692	0.633	**0.713**		
PDM	0.403	0.202	0.764	0.405	0.639	**0.772**	
WE	0.694	0.491	0.651	0.417	0.756	0.641	**0.708**

Bold values represent the square root of the average variance extracted (AVE) for each construct.

Furthermore, Pittino et al. (2018) applied the heterotraitmonotrait (HTMT) Ratio of correlations, which is an estimation method applied to determine the relationship between the components. A more recent test of discriminant validity in PLS-SEM is the HTMT. Figure 3 and Table 11 show that the differences between the HTMT values and the latent constructs are less than 1.0.

**Table 11:** Heterotrait-monotrait ratio.

Heterotrait-monotrait ratio (HTMT) (HTMT-90% Bootstrapped CI)	
AL < - > AC	0.656
AT <- > AC	0.676
AT <- > AL	0.350
EP < - > AC	0.318
EP < - > AL	0.186
EP < - > AT	0.487
OCB < - > AC	0.701
OCB < - > AL	0.429
OCB < - > AT	0.811
OCB < - > EP	0.693
PDM < - > AC	0.430
PDM < - > AL	0.193
PDM < - > AT	0.865
PDM < - > EP	0.448
PDM < - > OCB	0.694
WE <- > AC	0.791
WE <- > AL	0.488
WE <- > AT	0.781
WE <- > EP	0.483
WE <- > OCB	0.841
WE <- > PDM	0.761

Therefore, all the variables met the HTMT criteria of discriminant validity. According to Henseler et al. (2015), because the values are very low (less than 1), all the latent components have achieved discriminant validity.

All HTMT values fell below the 0.90 threshold (highest HTMT was PDM–Affective Trust at 0.865). To rigorously confirm discriminant validity, a bootstrapping procedure (5,000 samples) was conducted to evaluate HTMT_Inference. The 90% bias-corrected and accelerated confidence interval for PDM and Affective Trust ranged from 0.812 to 0.894, and for WE and OCB ranged from 0.718 to 0.821. Because no confidence interval contained 1.0, discriminant validity is statistically established (*p* < 0.05). Figure 6 shows the PLS Algorithmic Measurement Model.

**Figure 6:**
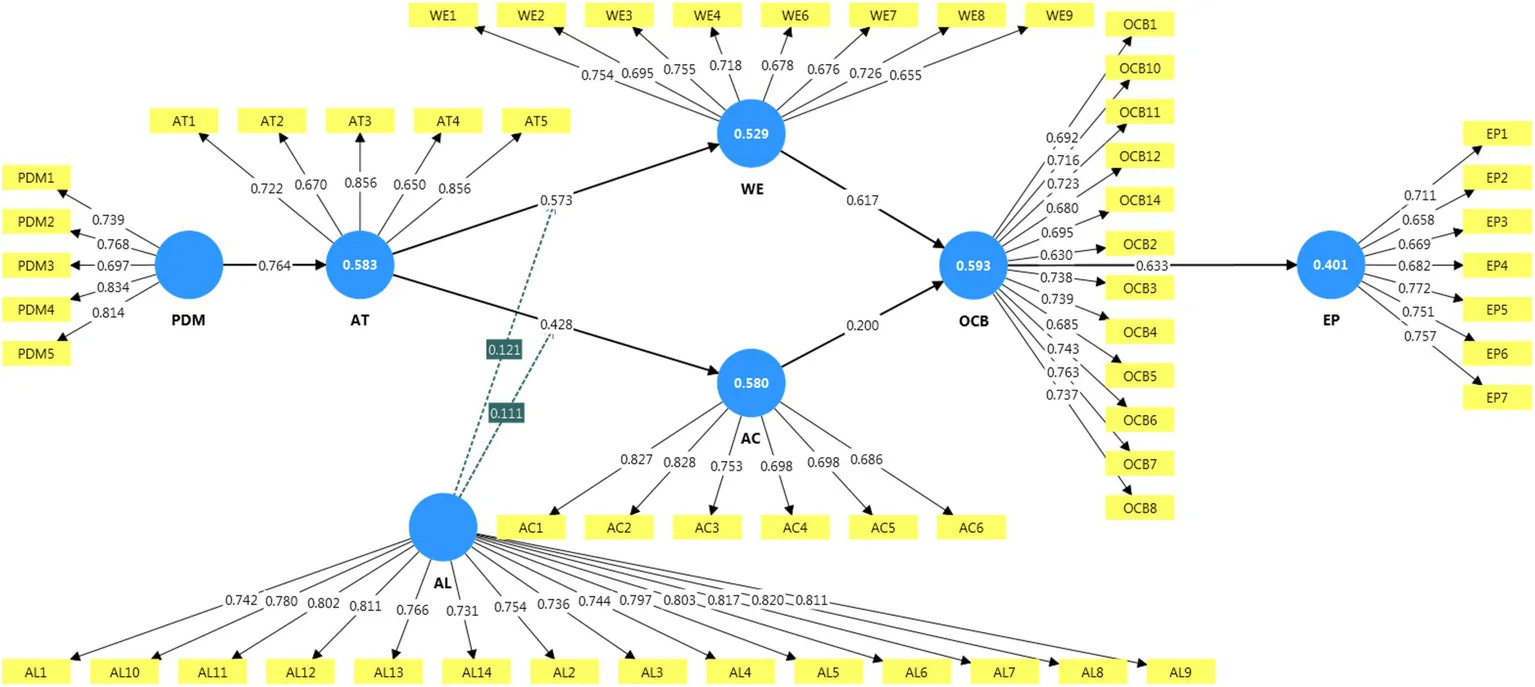
PLS algorithmic measurement model.

### 4.6 Structural modeling

The structural model is based on assessing four things: first, the significance of paths; second, the R-squared; third, the predictive relevance or impact of IVs on DV; and last, mediating and moderating effects.

#### 4.6.1 Direct relationships

Hair et al. (2013) have shown that significant paths provide statistical evidence of the model’s cause-and-effect relationships. The path coefficients can be compared to the beta values in regression line analysis, in which t-values indicate the significance of the path and the beta values indicate the regression coefficients (Henseler et al., 2009). The t-value of ±1.645 in a one-tailed test and ±1.967 or greater in a two-tailed test is considered significant (Hair et al., 2014). The structural model was evaluated by examining the significance of the hypothesized relationships. Figure 6 presents the standardized path coefficients, t-values, *p*-values, and bootstrapping results, while Table 12 summarizes the direct effects.

**Table 12:** Testing of hypotheses (direct effects from PLS).

**Hypotheses**	**Relationship**	**Beta**	**SE**	**t-Value**	***P*-value**	**Decision**
H1	PDM - > AT	0.764	0.021	36.572	0.000	Accepted
H2a	AT - > WE	0.573	0.036	15.935	0.000	Accepted
H2b	AT - > AC	0.428	0.033	12.991	0.000	Accepted
H3a	WE - > OCB	0.617	0.042	14.790	0.000	Accepted
H3b	AC - > OCB	0.200	0.051	3.940	0.000	Accepted
H5	OCB - > EP	0.633	0.035	18.107	0.000	Accepted
Constituent main effects						
	AL - > AC	0.530	0.028	18.680	0.000	
	AL - > WE	0.330	0.036	9.118	0.000	

The use of a structural model supports the proposed relationships. The involvement in decision making (PDM) has a substantial and significant impact on affective trust (*b* = 0.764, *p* < 0.001), which confirms the participative practices as a central significance relational cue within the context of which employees are inclined to interpret the benevolence of supervisors and build trust, which is in line with the Social Exchange Theory (SET) and is supported by the logic of Social Information Processing (SIP). In turn, affective trust is positively correlated with work engagement (*b* = 0.573, *p* = 0.001) and affective commitment (*b* = 0.428, *p* = 0.001), suggesting that trust is a background psychological resource that serves as a source of motivational-attitudinal reactions. These states further transform into discretionary behaviors, in which work engagement has a strong impact on organizational citizenship behavior (OCB; *b* = 0.617, *p* < 0.001), and affective commitment has a complementary but lower impact (*b* = 0.200, *p* = 0.001). Lastly, OCB has a significant relationship with employee performance (*b* = 0.633, *p* < 0.001), indicating that OCB is a proximal behavioral process upon which relational and motivational processes culminate in performance outcomes. Overall, the findings contribute to a SET-based association, in which participative decision-making triggers two-way interactions that are cognitively construed (SIP), internalized as trust and motivation, and eventually implemented in citizenship behaviors that lead to improved performance.

Constituent Main Effects: In accordance with recommended procedures for interaction models in PLS-SEM, direct paths from Authentic Leadership (AL) to Work Engagement (WE) and Affective Commitment (AC) were specified as constituent main effects. Including these main effects is necessary to prevent inflation of interaction terms and accurately assess the moderating influence of AL on the relationships between Affective Trust (AT) and employee attitudinal outcomes (WE and AC). So beyond the core hypotheses, the analysis also revealed significant direct paths from Authentic Leadership (AL) to employee attitudes, as AL is associated with AC (*β* = 0.530, *p* < 0.001) and WE (*β* = 0.330, *p* < 0.001). This suggests that Authentic Leadership serves as a “social cue” for employees. Even distinct from trust, the mere presence of an authentic leader fosters emotional attachment (AC) and work engagement (WE). Interestingly, AL is positively related to Commitment (*β* = 0.530), whereas Trust is positively related to Engagement. Figure 7 shows the bootstrapping for direct and indirect relationships along with *p*-values and R-squared.

**Figure 7:**
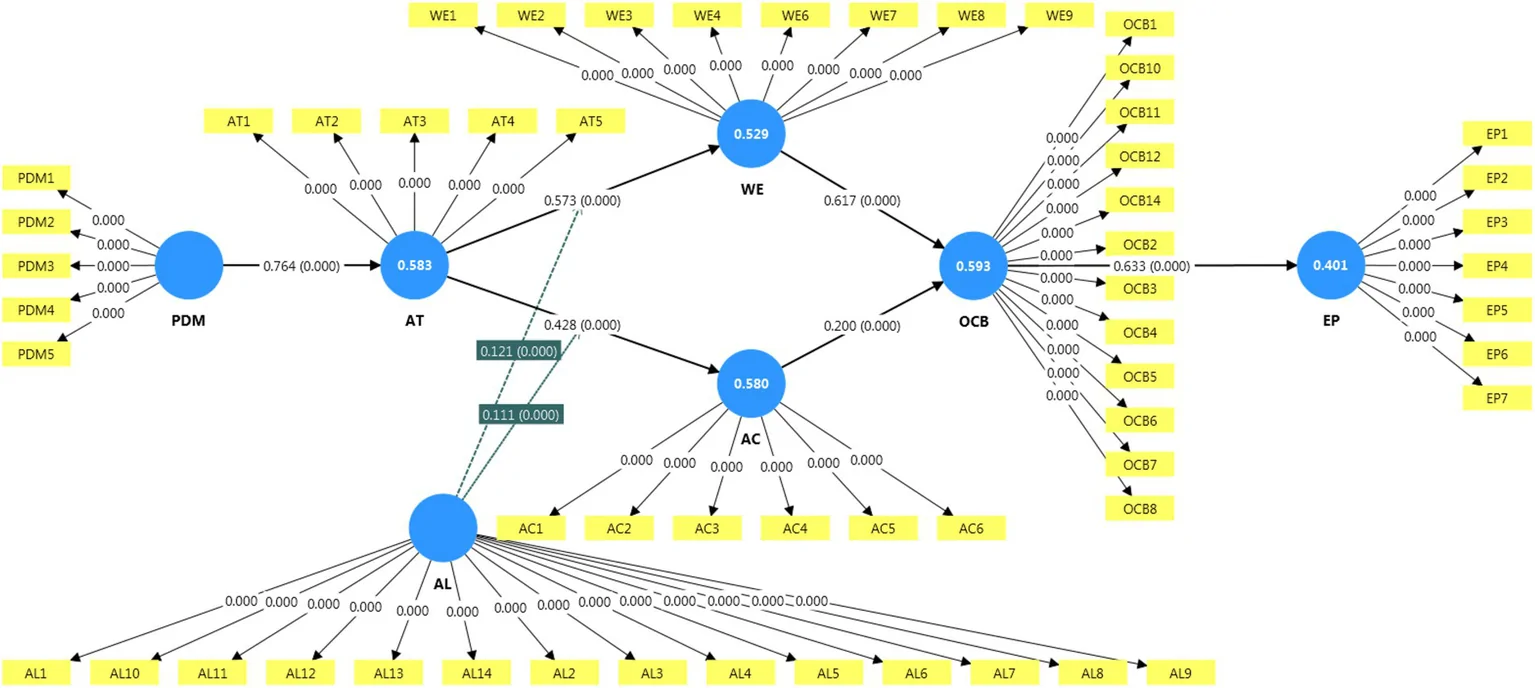
Bootstrapping for direct relationships along with *p*-value and *R*-squared.

#### 4.6.2 Significance of model

The next step was to analyze the hypotheses after establishing the path model’s suitability. The significance levels of the path coefficients were determined, and the predictive power of the structural model was evaluated using R-square values (Henseler et al., 2009). To assess the predictive power of the structural model, the coefficient of determination (*R*^2^) for each endogenous construct was analyzed. According to the rules of Hair et al. (2019) and Cohen (1988), *R*^2^ values of 0.75, 0.50, and 0.25 are substantial, moderately high, and weak, respectively. Table 13 shows the R-squared for each endogenous latent variable, indicating that the model exhibits a moderate to high degree of explanatory power. In particular, the hypothesized predictors explain 59.3 percent of the variance of Organizational Citizenship Behavior (OCB) (*R*^2^ = 0.593) and 58.3 percent of the variance of Affective Trust (AT) (*R*^2^ = 0.583). The model also explains 58.0 percent of the variance in Affective Commitment (AC) and 52.9 percent of the variance in Work Engagement (WE). Regarding the last dependent variable, the model explains 40.1 percent of the variation in Employee Performance (EP) (*R*^2^ = 0.401). All *R*^2^ values were significant (*p* < 0.001), indicating that the model explains the chosen employee outcomes well.

**Table 13:** R-square of endogenous latent constructs.

Constructs	R-Square
AT	0.583
AC	0.580
EP	0.401
OCB	0.593
WE	0.529

#### 4.6.3 Predictive relevance

The blindfolding method is also used in this study to evaluate the model’s predictive significance. The model has predictive relevance for the reflecting endogenous latent variable if the Q-square value is greater than zero (0), as recommended by Hair et al. (2014). Table 14 shows the Q-square values.

**Table 14:** Model’s predictive relevance (Q-square).

Constructs	*Q*-square	RMSE	MAE	Predictive relevance
EP	0.134	0.937	0.720	Modest
AT	0.580	0.652	0.491	High (Q^2^ > 0.50)
AC	0.495	0.714	0.547	Moderate-to-High
WE	0.523	0.693	0.544	High (Q^2^ > 0.50)
OCB	0.428	0.761	0.603	Moderate (Q^2^ > 0.25)

All *Q*^2^ values are generated via the PLSpredict procedure in SmartPLS 4.0 using 10-fold cross-validation. *Q*^2^ > 0 indicates predictive relevance.

In-sample predictive relevance was evaluated using the blindfolding procedure with an omission distance of *d* = 7. As detailed in Table 14, the cross-validated redundancy (*Q*^2^) values for all endogenous latent constructs are strictly greater than zero. Following Hair et al. (2021), these results confirm that the structural model possesses adequate in-sample predictive relevance across all endogenous variables. Specifically, Affective Trust (*Q*^2^ = 0.580), Work Engagement (*Q*^2^ = 0.523), Affective Commitment (*Q*^2^ = 0.495), and Organizational Citizenship Behavior (*Q*^2^ = 0.428) exhibiting high Q^2^ values. In contrast, Employee Performance (EP) yielded a comparatively lower *Q*^2^ value of 0.134 (RMSE = 0.937, MAE = 0.72). Following established PLS-SEM benchmarks (where *Q*^2^ values of 0.05, 0.25, and 0.50 indicate small, medium, and large predictive relevance), the predictive power for EP should be interpreted as modest in-sample predictive relevance. This reflects the reality that, while organizational citizenship behaviors and trust contribute meaningfully to performance, in-role employee performance in public-sector settings is also shaped by unobserved institutional and structural constraints. Moreover, in accordance with standard PLS-SEM conventions, because out-of-sample validation (PLSpredict) was not conducted, these findings are interpreted strictly in terms of theoretical in-sample predictive relevance rather than out-of-sample predictive accuracy.

Complementing the *R*^2^ values, the blindfolding cross-validated redundancy (*Q*^2^ = 0.134) confirms that the model possesses modest in-sample predictive relevance for Employee Performance (*Q*^2^ > 0). These metrics demonstrate that while the model successfully captures key relational and motivational drivers of performance, institutional constraints in public sector environments account for the remaining unexplained variance.

#### 4.6.4 Mediation and moderation of structural model

The interaction terms were estimated in SmartPLS 4 using the product indicator approach, and their significance was assessed through bootstrapping with 5,000 resamples. Bootstrapping has been used to investigate the mediation in this work as suggested by Preacher and Hayes (2004). To explore mediation and moderation, bootstrapping was first used to estimate path coefficients and then to compute t-values to assess the direct and indirect relationships between the variables. It was found that the variables are significantly related (see Table 15 and Figure 8). These are the following individual outcomes.

**Table 15:** Mediation analysis of the model.

**Hypotheses**	**Association**	**Sample mean (M)**	**SD**	***t*-value**	***P*-value**	**95% Bootstrapped CI [LL, UL]**	**Level of mediation**
H6 (Sequential mediator)	PDM - > AT - > WE - > OCB - > EP	0.171	0.021	7.986	0.000	(0.130, 0.212)	Full mediation
H7 (Sequential mediator)	PDM - > AT - > AC - > OCB - > EP	0.041	0.012	3.364	0.001	(0.017, 0.065)	Full mediation
Additional mediation paths							
	PDM - > AT - > AC	0.327	0.027	11.999	0.000		Partial mediation
	PDM - > AT - > WE	0.437	0.032	13.857	0.000		Partial mediation
	PDM - > AT - > WE - > OCB	0.270	0.028	9.658	0.000		Partial mediation
	PDM - > AT - > AC - > OCB	0.065	0.019	3.435	0.001		Partial mediation

CI = Confidence Interval (Bias-Corrected percentile); LL = Lower Limit; UL = Upper Limit. Bootstrap resamples = 5,000.

**Figure 8:**
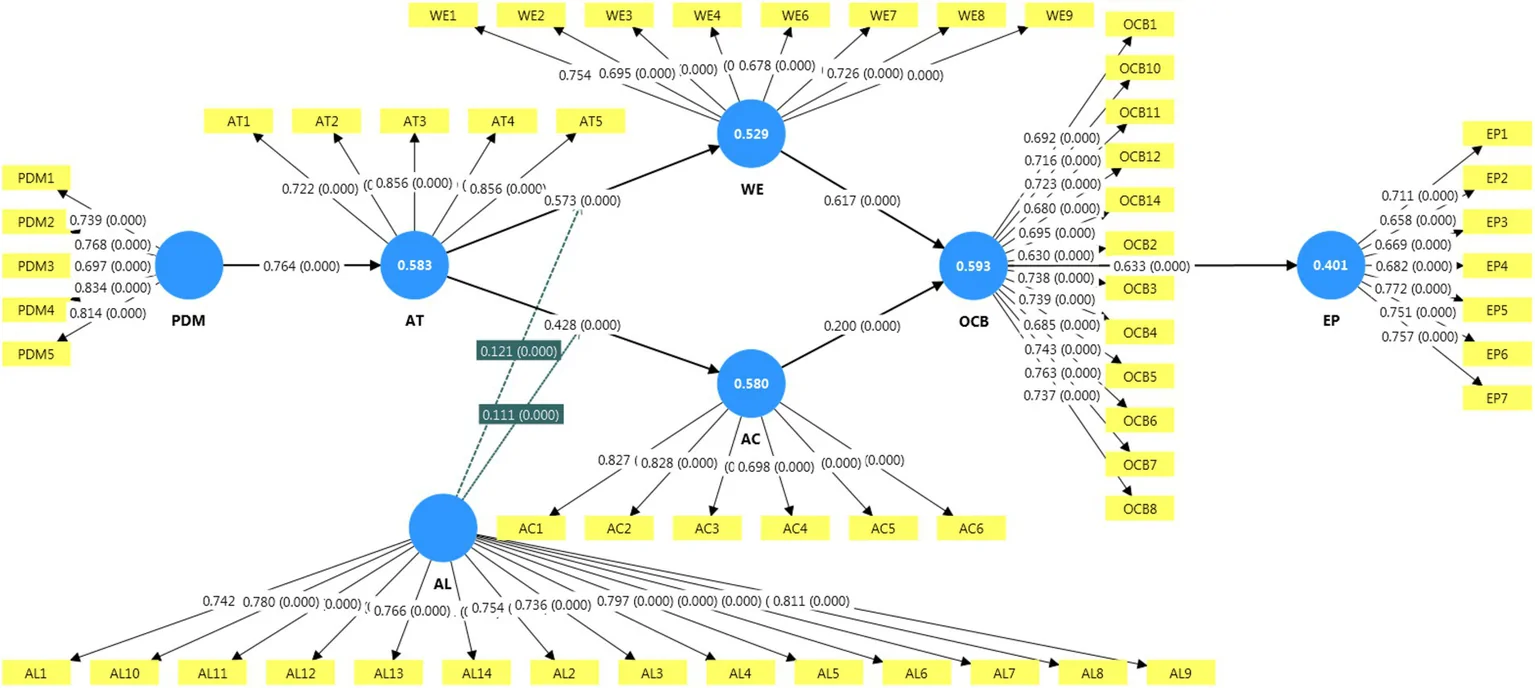
Direct and indirect relationships.

To assess the sequential mediation mechanisms, a bootstrapping procedure with 5,000 resamples was conducted in Smart-PLS 4.0 (Nitzl et al., 2016; Hair et al., 2021). As shown in Table 1, the specific indirect path via Work Engagement (PDM → AT → WE → OCB → EP) is statistically significant {*β* = 0.171, *t* = 7.986, *p* < 0.001, 95% CI (0.130, 0.212)}, supporting H6. Similarly, the specific indirect path via Affective Commitment (PDM → AT → AC → OCB → EP) is statistically significant {*β* = 0.041, *t* = 3.364, *p* = 0.001, 95% CI (0.017, 0.065)}, supporting H7. When testing the direct path from PDM → EP, the direct effect was non-significant (*β* = 0.000, *p* > 0.05), whereas the total indirect effect (*β* = 0.212, *p* < 0.001) equaled the total effect (*β* = 0.212, *p* < 0.001). This pattern establishes Full Mediation (Indirect-Only Mediation), confirming that PDM transmits its effect on employee performance indirect-only mediation through the sequential chain of affective trust, motivational states, and organizational citizenship behaviors.

Further mediation tests confirm that the mediating conduit through which the PDM is converted into greater engagement and commitment, which in turn triggers discretionary citizenship behaviors, is affective trust. The combination of these results supports a multi-stage social exchange process, with participative practices laying the foundation for trust, influencing employee perceptions and motivation, and eventually leading to performance-enhancing behaviors.

To test the moderating effects of Authentic Leadership (AL) on the relationships between Affective Trust (AT) and the two outcome variables (WE and AC), a two-stage approach with mean-centering was implemented in SmartPLS 4. The two-stage approach is preferred for continuous latent variable interaction in reflective models because it maximizes statistical power and minimizes collinearity compared to the product-indicator method (Hair et al., 2021). Significance was evaluated using bootstrapping with 5,000 resamples at a 95% bias-corrected confidence interval. The path coefficient for the interaction term (AL * AT → WE) was positive and statistically significant {*β* = 0.121, t = 5.177, *p* < 0.001, 95% CI (0.076, 0.166)}, supporting H4a. Similarly, the interaction term (AL * AT → AC) was positive and statistically significant {*β* = 0.111, t = 4.099, *p* < 0.001, 95% CI (0.058, 0.164)}, supporting H4b. To further interpret these moderation effects, simple slope analyses were conducted at three levels of Authentic Leadership: Low (−1 SD), Medium (Mean), and High (+1 SD), as summarized in Table 16.

**Table 16:** Moderating effects of the model.

**Dependent variable**	**Level of authentic leadership (AL)**	**Conditional effect (β)**	**Std. error**	***t*-value**	***p*-value**	**95% Bootstrapped CI [LL, UL]**
Work engagement (WE)	Low (−1 SD)	0.452	0.043	10.512	<0.001	(0.368, 0.536)
	Medium (Mean)	0.573	0.036	15.935	<0.001	(0.502, 0.644)
	High (+1 SD)	0.694	0.043	16.14	<0.001	(0.610, 0.778)
Affective commitment (AC)	Low (−1 SD)	0.317	0.043	7.372	<0.001	(0.233, 0.401)
	Medium (Mean)	0.428	0.033	12.991	<0.001	(0.363, 0.493)
	High (+1 SD)	0.539	0.043	12.535	<0.001	(0.455, 0.623)

For Work Engagement (WE), at low levels of AL (−1 SD), the simple slope of AT → WE was *β* = 0.452 (*p* < 0.001). At mean levels of AL, the effect was *β* = 0.573 (*p* < 0.001), and at high levels of AL (+1 SD), the effect increased to *β* = 0.694 (*p* < 0.001). For Affective Commitment (AC), at low levels of AL (−1 SD), the simple slope of AT → AC was *β* = 0.317 (*p* < 0.001). At mean levels of AL, the effect was *β* = 0.428 (*p* < 0.001), and at high levels of AL (+1 SD), the effect increased to *β* = 0.539 (*p* < 0.001). These interaction effects (AL × AT to AC and AL × AT to WE) suggest that trust can be more directly translated into motivational attachment and energetic involvement when workers perceive their leaders as genuine.

Figure 9 (AL * AT → WE), demonstrates that while Affective Trust positively influences Work Engagement across all levels, the positive slope is significantly steeper when Authentic Leadership is high (+1 SD), confirming a strengthening moderating effect.

**Figure 9:**
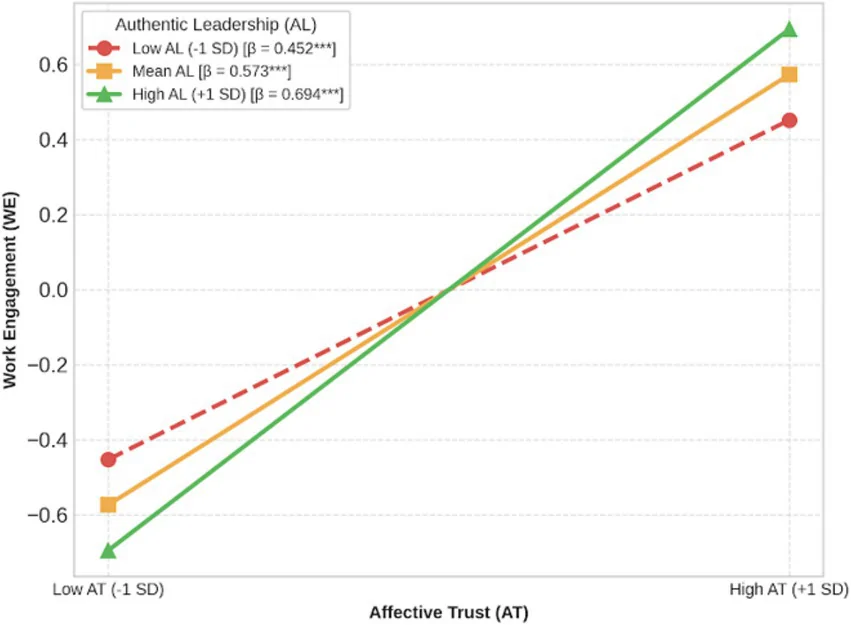
Moderation effect of (AL * AT → WE).

Figure 10 (AL * AT → AC), indicates that the positive impact of Affective Trust on Affective Commitment increases in magnitude as Authentic Leadership rises from low (−1 SD) to high (+1 SD).

**Figure 10:**
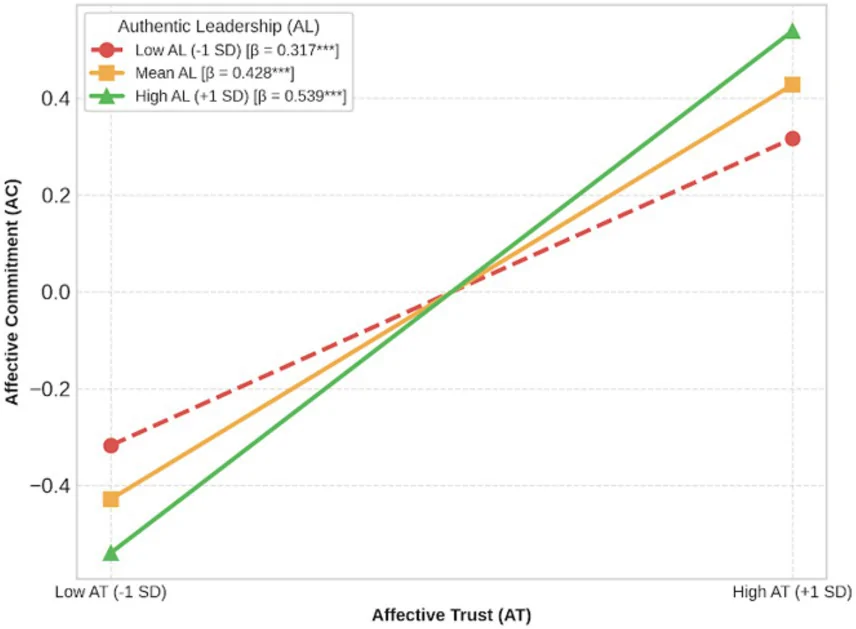
Moderation effect of (AL * AT → AC).

In line with the Social Exchange Theory, genuine leaders reinforce the norm of reciprocity by justifying employees’ trust in them. In contrast, the Social Information Processing theory proposes that authenticity is a salient social cue that shapes the interpretation and expression of trust. The conditional effects of interaction coefficients also means that trust correlates with commitment and engagement independently, though the degree of its influence is significantly conditional upon the genuineness of leadership cues.

## 5 Discussion

The key purpose of this study was to examine the mechanisms underlying employee performance in the Sierra Leonean public sector by integrating Social Exchange Theory (SET) and Social Information Processing (SIP) Theory. The findings support a sequential model in which Participative Decision-Making (PDM) is associated with Affective Trust, which is subsequently associated with Work Engagement and Affective Commitment and, ultimately, with Organizational Citizenship Behavior and employee performance. The findings further indicate that Authentic Leadership strengthens the relationships between Affective Trust and employees’ positive work attitudes. Consistent with SET (Blau, 1964), participation and authentic leadership may signal organizational support, respect, and positive exchange relationships. Although 86% of respondents held a university degree or higher, this demographic characteristic does not establish their expectations regarding workplace autonomy and should therefore be treated as contextual information. Similarly, PDM should be understood within the institutional conditions of Sierra Leone’s public sector, where formal administrative structures may influence opportunities for employee participation.

### 5.1 Discussion of results

The findings support the proposed relational pathway through which participative decision-making (PDM) is associated with employee performance. PDM was strongly associated with affective trust (*β* = 0.764, *p* < 0.001), while affective trust was positively related to work engagement (*β* = 0.573, *p* < 0.001) and affective commitment (*β* = 0.428, *p* < 0.001). Consistent with Social Exchange Theory and Social Information Processing Theory, these results suggest that participation may function as a social signal that is associated with stronger relational confidence and subsequent positive employee attitudes.

The two motivational routes subsequently converged in organizational citizenship behavior. Both work engagement (*β* = 0.617, *p* < 0.001) and affective commitment (*β* = 0.200, *p* < 0.001) were positively associated with OCB, which in turn was strongly related to employee performance (*β* = 0.633, *p* < 0.001). The estimated coefficient for work engagement was larger than that for affective commitment; however, because their difference was not formally tested, this finding should be interpreted as a difference in estimated magnitude rather than evidence that one pathway is statistically stronger. Similarly, the sequential indirect effects through work engagement (*β* = 0.171, *p* < 0.001) and affective commitment (*β* = 0.041, *p* = 0.001) were both significant, with the former having the larger estimated effect.

Authentic leadership further shaped the trust-to-attitude relationships. It strengthened the associations of affective trust with both work engagement (*β* = 0.121, *p* < 0.001) and affective commitment (*β* = 0.111, *p* < 0.001). This finding suggests that the relational value of trust may depend partly on how employees interpret the authenticity and credibility of their leaders, extending the SET–SIP explanation beyond direct relationships.

Importantly, these findings should not be interpreted as establishing a fixed causal sequence. The cross-sectional design permits alternative ordering, including the possibility that employees who perceive themselves as high performers subsequently report greater trust, engagement, commitment, or OCB. The use of self-reported measures also leaves room for halo effects, social desirability, and shared-source variance. Although the measurement analysis supported discriminant validity, the relatively strong association between work engagement and OCB (r = 0.758; HTMT = 0.841) indicates that their conceptual and perceptual proximity should be acknowledged.

Finally, contextual interpretations require caution. The relatively high educational attainment of respondents, digital access, or bureaucratic characteristics may help contextualize the findings, but these factors were not directly tested and therefore should not be presented as empirical explanations. Overall, the evidence supports a relational–motivational–behavioral pathway linking PDM with performance, while leaving causal ordering and the relative distinctiveness of some mechanisms open for longitudinal and multi-source investigation.

### 5.2 Theoretical implications

This study extends the application of Social Exchange Theory (SET) and Social Information Processing (SIP) Theory to the Sierra Leonean public-sector context. The findings indicate that participative decision-making (PDM) may be associated with employee performance through a sequential relational, motivational, and behavioral pathway involving affective trust, work engagement, affective commitment, and organizational citizenship behavior (OCB). The strong PDM–affective trust relationship provides contextual support for SET by suggesting that participation may signal organizational support, respect, and inclusion. The findings also complement SIP theory by indicating that participative practices and authentic leadership may provide social cues that shape employees’ attitudes. The significant sequential mediation further suggests that trust, motivation, and discretionary behavior may jointly explain how PDM is associated with performance. Overall, the study provides contextual evidence supporting the relevance of SET and SIP in an underexplored public-sector setting, while offering a basis for future research across different institutional contexts.

### 5.3 Practical implications

The findings provide context-sensitive implications for public-sector managers and HR practitioners. Organizations may consider strengthening participative mechanisms, including employee consultation, feedback, and involvement in relevant work-related decisions, where institutional conditions permit. Organizations may also consider leadership practices emphasizing transparency, ethical conduct, openness, and consistency, as authentic leadership strengthened the relationships between affective trust and employee attitudes. Similarly, initiatives supporting employee engagement, recognition, professional development, and constructive citizenship behaviors may be considered as part of broader employee-management strategies. Overall, the findings suggest that public-sector organizations may benefit from an integrated approach combining participation, authentic leadership, employee engagement, and supportive work conditions, with implementation adapted to organizational resources, structures, and institutional requirements.

### 5.4 Limitations and future research

While this study provides robust insights and adheres to rigorous methodological standards, it contains certain limitations that present valuable avenues for future research. *First*, this study utilized a cross-sectional research design, capturing data at a single point in time. While the theoretical sequencing of the multistage model is strongly supported by SET and SIP, definitive causal inferences cannot be drawn. While the sequential mediation model presented in this study is theoretically meaningful and deeply grounded in Social Exchange Theory and Social Information Processing theory, the cross-sectional nature of the data cannot fully confirm hypothesized theoretical ordering or establish absolute causality. Future research should employ longitudinal or cross-lagged panel designs to observe how the introduction of participative practices influences the evolution of affective trust, engagement, and performance over time. Future research should employ longitudinal or time-lagged research designs to empirically validate the hypothesized theoretical sequence of these psychological and behavioral constructs. *Second*, reliance on self-reported survey data introduces the potential for common method variance (CMV), particularly for behavioral and performance outcomes. Although procedural and statistical remedies (e.g., assessing discriminant validity) were applied to mitigate this, future studies could benefit from multi-source data collection, incorporating supervisor ratings for Employee Performance and peer assessments for Organizational Citizenship Behavior, to increase the objectivity of the measurement of the outcome variables. Thirdly, this study had an organizational context restricted to the public sector of Sierra Leone. Although this helps bridge the crucial gap in Western bias in organizational research, it restricts the direct generalizability of the results to other contexts. The multistage approach will need to be applied across private enterprises and varied cultural settings to confirm the theory’s universality in the future. Fourthly, the research has been limited to the study of affective trust and authentic leadership only. There are many complications in the business world, and other forms of trust, such as cognitive trust, or leadership styles like transformational, servant, or inclusive leadership, can yield different results. To compare results, future research can include these types of variables in their models. Finally, the use of non-probability, convenience sampling has lowered the external validity of this research, as it can be biased and introduces selection bias in the samples used. Probability methods, such as stratified random sampling, can be used to increase representativeness. *Finally*, a notable limitation of this study is the reliance on self-reported, subjective survey data. As already stated, when measuring psychological constructs like Affective Trust and Work Engagement, self-reporting is inevitable, as these are intrapsychic constructs. However, when measuring Employee Performance with subjective measures, there is a likelihood of common method variance (CMV) and social desirability bias. Although discriminant validity was established using statistical techniques, future studies in public administration should use objective data sources, such as civil service performance ratings or citizen satisfaction surveys.

## 6 Conclusion

This study examined the relationships among participative decision-making, affective trust, work engagement, affective commitment, organizational citizenship behavior, authentic leadership, and employee performance in Sierra Leone’s public sector. The findings showed that participative decision-making was positively associated with affective trust, which was subsequently associated with work engagement and affective commitment. Both were positively associated with organizational citizenship behavior, which in turn was positively related to employee performance. The sequential indirect effects through work engagement and affective commitment were also significant, while authentic leadership strengthened the relationships between affective trust and both employee attitudes. These findings provide contextual evidence supporting the application of Social Exchange Theory and Social Information Processing Theory in Sierra Leone’s public-sector context. However, the cross-sectional design, convenience sampling, and self-reported measures limit causal inference and leave open alternative explanations, including reverse ordering and common-source effects. Future research should therefore employ longitudinal, multisource, and objective performance measures to further examine these relationships and their temporal ordering.

## Data Availability

The raw data supporting the conclusions of this article will be made available by the authors, without undue reservation.
